# Rab24 protein levels show dynamic changes in mouse tissues and human cancers

**DOI:** 10.1007/s00441-025-04043-4

**Published:** 2026-01-27

**Authors:** H. G. Mauricio Ramm, Farhad Ahmed, Sadaf Fazeli, Matthieu Bourgery, Martin Alexander Lopez, Lav Tripathi, Ilmo Leivo, Pernilla Syrjä, Eeva-Liisa Eskelinen

**Affiliations:** 1 Institute of Biomedicine, University of Turku, Kiinamyllynkatu 10, 20520 Turku, Finland; 2 Department of Medicine Huddinge, Center for Hematology and Regenerative Medicine, Karolinska Institute, Huddinge, Sweden; 3 Institute of Clinical Molecular Biology, Kiel University, Kiel, Germany; 4 Department of Internal Medicine I, University Hospital Schleswig-Holstein, Campus Kiel, Kiel, Germany; 5 Institute of Biomedicine, Pathology, University of Turku, Finland, and Turku University Hospital, Turku, Finland; 6 Section of Veterinary Pathology and Parasitology, Department of Veterinary Biosciences, University of Helsinki, Helsinki, Finland

**Keywords:** Rab24, Mouse tissues, Cancer, Neuroblastoma, Brain cancer, Pancreatic neuroendocrine tumours

## Abstract

Rab24 is an unusual member of the Rab family of small GTPases, implicated in autophagy, endocytosis and cell division. In order to elucidate possible organ and age-specific roles of Rab24, we investigated tissue-specific levels of Rab24 in mice by western blotting and immunohistochemistry from postnatal day one to 9 months of age. In adult mice, the highest protein levels were found in the brain followed by the kidney, whereas lower levels were detected in the pancreas, spleen, liver, lung, heart, and skeletal muscle. Dynamic changes in Rab24 levels were observed during early postnatal development, with a sharp increase in the brain at postnatal day 14, after which the level remained high into adulthood. In the heart, skeletal muscle, pancreas and liver, higher Rab24 levels were observed during the first two postnatal weeks, after which the levels dropped and stayed low until adulthood. The age-dependent changes suggest age- and organ-specific regulation of Rab24 protein levels and possible organ-specific roles for Rab24 in development and maturation. Immunohistochemistry of the brain revealed that Rab24 was mostly present in neuronal cells in 1-month-old and older mice. Also, epithelial cells in several tissues showed high Rab24 levels. These results suggest possible roles for Rab24 in neuronal and epithelial maintenance. We further analysed immunohistochemical staining for RAB24 in human cancers and normal tissues. RAB24 staining in cancers of the breast and skin was higher than in the corresponding normal tissues, while it was reduced in cancers of the digestive system and the urinary tract. We also observed elevated RAB24 staining in medulloblastoma and neuroblastoma, two paediatric cancers of neuronal origin. In pancreatic neuroendocrine tumours that originate from islet cells, RAB24 levels were lower than in normal pancreatic islet cells. Collectively, our findings provide a comprehensive overview of RAB24 protein levels across mouse tissues and a wide spectrum of human cancers. The observed differences in RAB24 levels between cancer types and between malignant and normal tissues, suggest that RAB24 may serve as a potential diagnostic or differentiation marker in specific tumour types.

**Supplementary Information:**

The online version contains supplementary material available at 10.1007/s00441-025-04043-4.

## Introduction

Rab GTPases regulate intracellular membrane trafficking events ranging from vesicle formation, vesicle transport, and membrane tethering to membrane fusion. Rab24 was first described in 1993 to localize to the endoplasmic reticulum, Golgi apparatus and late endosomes (Olkkonen et al. 1993). It is an unusual member of the Rab family due to the presence of an atypical amino acid in the GTP-binding region (Erdman et al. 2000).

We and others showed that Rab24 functions in autophagy (Munafo and Colombo 2002; Yla-Anttila et al. 2015), a catabolic process that recycles organelles and aggregate-prone proteins by transporting them to lysosomes, thereby producing substrates for biosynthesis and energy production. Degradative autophagic vacuoles accumulated in Rab24-knockdown cells, suggesting a block at the late stages of the autophagy pathway (Yla-Anttila et al. 2015). Further, the Q38P point mutation in Rab24, leading to degeneration of cerebellar Purkinje neurons, was identified as the cause of canine hereditary ataxia in Gordon setters and Old English sheepdogs (Agler et al. 2014). The affected canine neurons accumulate autolysosomes and ubiquitin-protein aggregates, suggesting a defect in the autophagy pathway, in agreement with our results on the role of Rab24 in autophagy (Yla-Anttila et al. 2015). More recently, a G80V point mutation in Rab24 was also reported to cause cerebellar ataxia in dogs (Schwarz et al. 2025). Further, Rab24 was shown to regulate endosomal degradation by interacting with the late endosomal protein Rab7 (Amaya et al. 2016). Together, these results show that Rab24 is important for neuronal health, possibly by regulating the delivery of autophagic and endocytic cargo to lysosomes.

In humans, RAB24 has been associated with fatty liver disease and hepatocellular carcinoma (HCC). Liver RAB24 levels positively correlate with body fat and are highly increased in the livers of obese patients with non-alcoholic fatty liver disease (NAFLD; Seitz et al. 2019). Rab24 knockdown in mouse liver enhanced autophagic flux and mitochondrial connectivity and reduced hepatic fat and serum cholesterol levels in obese mice, confirming the link between Rab24 levels and liver fat accumulation. Moreover, Rab24 was also shown to regulate mitochondrial fission and activation in hepatocytes. Thus, Rab24 regulates mitochondrial activation in the liver, which has a direct impact on hepatic and systemic energy homeostasis (Seitz et al. 2019).

Furthermore, several studies have shown RAB24 to be overexpressed, and/or associated with poor prognosis in several cancers, particularly in liver cancer (Chen et al. 2017b; Gu et al. 2025; He et al. 2002; Yang et al. 2020; Zhang et al. 2020; Zhu et al. 2020). RAB24 expression is increased in HCC due to downregulation of microRNA miR-615-5p, which suppresses RAB24 expression (Chen et al. 2017b). Ectopic overexpression of RAB24 enhanced the malignant phenotype of HCC cells by promoting cell motility, invasion and adhesion, by accelerating cell cycle progression, reducing apoptosis, and by facilitating epithelial to mesenchymal transition, while knockdown of RAB24 had opposite effects (Chen et al. 2017b). These findings show that RAB24 plays a significant role in promoting the malignant phenotype of HCC cells. Furthermore, high RAB24 expression is an unfavourable prognostic marker in prostate cancer (Hu et al. 2020). On the contrary, RAB24 was reported to be an independent low-risk factor in pancreatic adenocarcinoma (Deng et al. 2022). According to the Human Protein Atlas (proteinatlas.org, Uhlen et al. 2015), RAB24 is classified as a potentially favourable marker in clear cell renal cell carcinoma and an unfavourable marker in glioblastoma. These findings highlight RAB24’s context-dependent prognostic value in different cancer types.

Despite these advances, the expression patterns of Rab24 in different tissues and developmental stages have not been analysed. Knowledge on these patterns can provide insights into Rab24’s possible physiological roles and potential contributions to age-related diseases. Given that mice are widely used as model organisms in autophagy research, and that Rab24 plays a role in autophagy, understanding the normal age-dependent expression patterns of Rab24 protein is important, as such patterns may serve as potential confounding factors when interpreting experimental results.

In this study, we analysed Rab24 protein levels in the brain, heart, liver, lung, kidney, spleen, skeletal muscle, and pancreas of C57BL/6NCrl mice in different age groups from postnatal day 1 to 9 months using western blotting. Immunohistochemistry (IHC) was also performed to identify the specific cell types expressing Rab24 protein in these tissues. Our results revealed distinct cell- and tissue-specific patterns and dynamic, age-dependent changes in Rab24 levels. This is the first study to provide a comprehensive record of Rab24 protein levels in mouse tissues and cell types according to age. In addition, we analysed tissue microarrays (TMAs) from various human cancers and found that in many cancers, RAB24 levels differ from the corresponding normal tissues. RAB24 staining in cancers of the breast and skin was higher than in the corresponding normal tissues, while it was reduced in cancers of the digestive system and the urinary tract. Further, we report RAB24 protein overexpression in medulloblastoma and neuroblastoma, two cancers originating from neuronal cells, and decreased RAB24 protein expression in pancreatic neuroendocrine tumours (PNETs) originating from pancreatic islets. Our findings provide a basis for further studies regarding the role of RAB24 as a prognostic or predictive biomarker in cancer.

## Material and methods

### Ethical compliance

All experimental procedures involving animals were ethically reviewed and approved by the Project Authorization Board of the Regional State Administrative Agency of Southern Finland (ESAVI/613/2019), and complied with the guidelines of the Directive 2010/63/EU of the European Union.

Sections from human multicancer TMAs and PNET TMAs were obtained from Helsinki Biobank, following approval by the Ethics Committee of the Hospital District of Helsinki and Uusimaa (HUS/697/2020). No information on patient identity, tumour stage or patient survival was delivered with the TMAs. The samples in the Helsinki Biobank are stored after receiving informed consent from all patients. Neuroblastoma and brain cancer TMAs were obtained from Tissue Array (TissueArray.Com, USA). The TMAs were delivered without any direct identifiers, ensuring that individuals could not be re-identified.

The study conformed to the standards of the Declaration of Helsinki (World Medical Association, 2013). According to Paragraph 32 of the Declaration of Helsinki, secondary research involving completely anonymized data in non-interventional contexts does not require ethical review or informed consent.

### Preparation of mouse tissue extracts and immunoblotting

Mice were group-housed under controlled temperature and a 12-h light–dark cycle with same-sex littermates, and given free access to food and water. C57BL/6NCrl mice of different ages (1 day, 7 days, 14 days, 1 month, 3 months, 6 months, and 9 months) were sacrificed by cervical dislocation (1, 7, and 14-day-old animals) or by carbon dioxide asphyxiation (1 month and older animals). Four mice, two males and two females, were used for each age group. Samples from the cerebral cortex, heart, liver, lung, kidney, spleen, skeletal muscle (*tibialis anterior*), and pancreas were collected and snap-frozen in liquid nitrogen. For protein extraction, approximately 35 mg of tissue was homogenized in 140 µl of homogenization buffer (50 mM Tris–HCl, pH 7.4, 10 mM NaCl, 1% NP-40, and 1 mM EDTA) supplemented with protease and phosphatase inhibitors (A32959, Thermo Scientific). After adding a 5-mm stainless steel bead (69989, Qiagen), the samples were lysed using a TissueLyser LT (85600, Qiagen) at 50 Hz for 3 min. Subsequently, an additional 140 µl of homogenization buffer was added, bringing the total buffer volume to 280 µl per 35 mg of tissue. The lysates were rotated end-over-end at +4 °C for 1 h and centrifuged at +4 °C, 16,000 g for 15 min. Protein concentration of the supernatants was determined by bicinchoninic acid (BCA) assay (23228, Thermo Scientific), and SDS sample buffer (100 mM sodium phosphate, pH 7.5, 2% w/v SDS, 10% v/v glycerol, 5% v/v β-mercaptoethanol, 0.004% w/v bromophenol blue) was then added. The samples were heated at +95 °C for 4 min and stored at −20 °C.

Per lane, 10 µg of total protein was resolved on a 12% SDS-PAGE gel and blotted onto a polyvinylidene difluoride (PVDF) membrane (88518, Thermo Scientific). Total proteins were stained using TotalStain Q (AC2225, Azure Biosystems) and the blots were imaged using Azure Sapphire imaging system (Azure Biosystems). For antibody staining, the membranes were blocked with 5% non-fat milk powder in Tris-buffered saline (100 mM Tris–HCl, pH 7.6, 1.5 M NaCl) containing 0.05% Tween-20 (TBS-T). Membranes were probed with affinity-purified rabbit anti-Rab24 (11445–1-AP, Proteintech) or with mouse anti-GAPDH (ab8245, Abcam) in blocking solution at +4 °C overnight. Membranes were washed, incubated with horseradish peroxidase (HRP)-conjugated anti-rabbit IgG secondary antibody (111–035-003, Jackson ImmunoResearch) at room temperature for 1 h and the bands were visualized with Clarity™ Western ECL Substrate (1705061, Bio-Rad). Blots were imaged using an Azure Sapphire imaging system (Azure Biosystems), and the bands were quantified using Fiji/ImageJ (Schindelin et al. 2012). Details of all antibodies used in this study are listed in Table S1. To compare Rab24 levels between different blots, tissue extract from one 1-month-old liver was used as a control sample in the blots. The Rab24 signals were first normalized to total protein and then normalized to the control sample in each gel.

### Preparation of mouse tissues for immunohistochemistry

Immunohistochemistry was performed for C57BL/6NCrl mice aged 7 days and 1, 3, and 6 months. Four 7-day-old mice, three males and one female, were used. Eight mice each, four males and four females, were used for the age groups 1, 3, and 6 months. Cervical dislocation followed by decapitation was used for euthanasia of 7-day-old mice. Older mice (1, 3, and 6 months) were anesthetized by intraperitoneal injection of ketamine (75 mg/kg) and xylazine (10 mg/kg), and transcardially perfused with phosphate-buffered saline (PBS), pH 7.4, followed by 4% paraformaldehyde in PBS, using a peristaltic pump at a flowrate of 5 ml per minute. For all age groups, samples from the brain, heart, liver, lung, kidney, spleen, skeletal muscle, and pancreas were collected and post-fixed in 4% paraformaldehyde in PBS at +4 °C for 24 h. The samples were dehydrated, embedded in paraffin, and 5-µm sections were cut and mounted on glass slides.

### Immunohistochemical staining

For IHC, tissue sections were deparaffinized in xylene and rehydrated in a graded ethanol series. Endogenous peroxidases were quenched with 3% H_2_O_2_ in methanol for 20 min. Antigen retrieval was performed in 10 mM citrate buffer, pH 6.0, by microwave heating at 640 W for 7 min and at 480 W for 7 min. Sections were permeabilized in 0.1% Triton X-100 in TBS-T for 5 min. Blocking was performed with 5% normal goat serum in TBS at room temperature for 1 h. Sections were incubated in affinity-purified rabbit anti-Rab24 (11445–1-AP, Proteintech), rabbit anti-LC3 (NB-100-2331, Novus), rabbit anti-Rab5 (sc-28570, Santa Cruz), rabbit anti-Rab7 (9367S, Cell Signaling), rabbit anti-p62/SQSTM1 (AP2183b, Abgent), or a mixture of rat anti-LAMP1 (1D4B, Developmental Studies Hybridoma Bank/DSHB) and rat anti-LAMP2 (ABL-93, DSHB) in 5% normal goat serum at +4 °C overnight (Table S1). To enhance the weak signals, antibodies against LAMP1 and LAMP2 were applied together on the same sections, as both proteins colocalize on lysosomal membranes. The next day, the sections were washed and incubated in biotinylated goat anti-rabbit IgG included in Vectastain Elite ABC-HRP Kit (PK-6101, Vector Laboratories) or HRP-conjugated goat anti-rat IgG (112–035-003, Jackson Laboratory) at room temperature for 1 h. Sections were washed, and those labelled with the biotinylated secondary antibody were incubated with an avidin horseradish peroxidase complex (PK-6101, Vector Laboratories) in PBS at room temperature for 40 min. All sections were then washed and incubated in 3,3’-diaminobenzidine (DAB, ready-made reagent, SK-4100, Vector Laboratories) for 1 min. Slides were counterstained with Mayer’s haematoxylin (105.3 mM aluminium potassium sulphate, 3.308 mM haematoxylin, 505.3 µM sodium iodate, 4.758 mM citric acid, and 302.2 mM chloral hydrate) for 1 min, dehydrated in a graded alcohol series and mounted using Pertex® mounting medium (00811, HistoLab).

To confirm the specificity of the immunostaining, serial sections of the tissues were stained both with the protocol described above, and with a control protocol in which the primary antibody incubation was replaced by a prolonged blocking step (Fig. S1). The same control staining was performed for the PNET sections (Fig. S2).

For Ki-67 IHC staining, TMA sections were deparaffinized in xylene and rehydrated in a graded ethanol series. Antigen retrieval was performed in 10 mM citrate buffer, pH 6.0, at +99**°**C for 20 min in a PT Module (Lab Vision™). Slides were set in a Shandon™ Coverplate system. Endogenous peroxidases were quenched in 3% H_2_O_2_ in PBS for 10 min, and blocking was performed with 20% normal goat serum in PBS at room temperature for 20 min. Sections were incubated in rabbit monoclonal anti-Ki-67 (RM-9106-S1, Thermo Scientific) in 1% BSA in PBS at room temperature for 60 min. Subsequently, the sections were washed and incubated in biotinylated goat anti-rabbit IgG (BA-1000, Vector Laboratories) in PBS at room temperature for 30 min. Sections were washed, incubated with an avidin-HRP complex (PK-6100, Vector Laboratories) in PBS at room temperature for 30 min, washed again and incubated in DAB (BS04, ImmunoLogic a WellMed Company) for 6 min. Slides were counterstained with Harris haematoxylin (1.09253, Sigma-Aldrich), dehydrated in a graded alcohol series and mounted using Pertex® mounting medium. A control slide was incubated without the primary antibody.

Images of whole slides were acquired using a Pannoramic 250 Flash slide scanner equipped with a 20 × objective (3DHistech). Images were analysed and cropped using CaseViewer (3DHistech) and Fiji/ImageJ software (Schindelin et al. 2012).

### Analysis of human tissue microarrays

Paraffin sections from TMAs containing samples from 75 different types of human cancers belonging to 220 patients (Table S2), and from TMAs containing human pancreatic PNET samples from 122 patients (Table S3) were obtained from Helsinki Biobank. Part of the tissue cores also contained normal tissues that had been removed together with the tumours, or separate tissue cores containing normal tissue. Sections from TMAs containing brain cancer and brain tissue samples from 104 patients (CNS2081a, TissueArray.Com, Table S4) and neuroblastoma and peripheral nerve tissue samples from 30 patients (NB642d, TissueArray.Com, Table S5) were also used. The sections were stained as described above.

Quantification of RAB24 staining intensity and Ki-67 labelling index was performed using QuPath software version 5.0.1 (Bankhead et al. 2017). Whole-slide images were opened in QuPath and set to the H-DAB image type. A TMA grid was defined using the *TMA dearrayer* function. For the analysis of RAB24 staining intensity, RAB24-positive cells were detected and classified using the *positive cell detection* function. Tissue folds and other artifacts were manually excluded. In PNET samples, tumour tissue, pancreatic islets, and connective tissue were manually annotated in 15–20 representative areas, and the *train object classifier* function was used to classify the remaining tissues across the entire TMA. QuPath categorized the RAB24 staining intensity on a scale from 0 to 3, where 0 indicated no staining, 1 + weak staining, 2 + moderate staining, and 3 + strong staining. Each category had a separate threshold for the RAB24 staining intensity; the values for the threshold were selected manually using scores given by a pathologist as reference. RAB24 staining intensity was then quantified using the H-score, calculated as: H-score = 0 × (% negative cells, 0) + 1 × (% weakly positive cells, 1 +) + 2 × (% moderately positive cells, 2 +) + 3 × (% highly positive cells, 3 +). The H-score data were exported using the *show TMA measurement* function.

For the determination of Ki-67 index, whole-slide TMA images were processed as above in QuPath. A TMA grid was defined, and Ki-67-positive nuclei were detected using the *positive cell detection* function. Nuclei located in tissue folds were manually excluded. Tumour tissue and connective tissue were manually annotated in 10–20 representative areas in randomly selected cores, and an object classifier was trained to classify tissues throughout the TMA. The percentage of Ki-67-positive nuclei in tumour tissue was exported using the *show TMA measurement* function.

### Cell culture

Neuro-2a cells (CCL-131, ATCC) were grown in MEMα (L0476, Biowest) supplemented with 10% foetal bovine serum (11573397, Gibco), 1% non-essential amino acids (11140050, Gibco), 2 mM glutamine (ECB3004D, EuroClone), and 1% penicillin–streptomycin. HeLa cells (CCL-2, ATCC) were grown in Dulbecco’s modified Eagle medium (DMEM, 41965039, Gibco) supplemented with 10% foetal bovine serum, 2 mM glutamine, and 1% penicillin–streptomycin. Both cell lines were grown at +37 °C in 5% CO_2_.

### Generation of Rab24-knockout cells

Stable Rab24 knockout (KO) Neuro-2a and HeLa cell lines were generated using CRISPR-Cas9 genome editing. Single guide RNA (sgRNA) targeting the mouse or human Rab24 gene were designed using the CHOPCHOP web tool (Labun et al. 2016, 2019; Montague et al. 2014). For each construct, two sgRNAs were selected, chosen for efficiency and low predicted off-target activity (Table S6), and used as double-stranded DNA fragments (gBlocks Gene Fragments, Integrated DNA Technologies). The sgRNAs were cloned into the pSpCas9(BB)−2A-GFP (PX458) expression vector via the BbsI restriction site, allowing co-expression of Cas9, GFP, and both sgRNAs from a single plasmid.

Cells were transfected with the PX458-sgRNA constructs using PolyFect transfection reagent (301107, Qiagen) and GFP-positive cells were isolated by single-cell sorting into 96-well plates using a SH800 cell sorter (Sony). Individual clones were expanded and screened for deletion of the targeted genomic region (Fig. S3a) by PCR amplification of genomic DNA (Fig. S3b). Loss of Rab24 protein expression in knockout clones was validated by western blotting (Fig. S3c). The knockout generation and screening strategy (Fig. S3a), the selected sgRNAs (Table S6), and sequencing primers for PCR screening (Table S7) are shown in the supplementary materials.

### Cell proliferation and migration assays

Cell proliferation and scratch-wound assays were performed using wild-type (WT) and Rab24 KO Neuro-2a (clone A), and HeLa cell lines. For scratch-wound assays, 1.2 × 10^5^ Neuro-2a cells or 6 × 10^4^ HeLa cells were seeded per well in 96-well plates (130188, Thermo Scientific). After 24 h, a scratch wound was introduced in each well using a WoundMaker™ (4563, Sartorius). The plates were placed into an IncuCyte S3 live-cell imaging system (Sartorius), and images were acquired at 12-h intervals for up to 96 h (Neuro-2a) or 72 h (HeLa). Relative wound density was quantified using IncuCyte 2024B software (9600–0012, Sartorius). Scratch-wound assays were conducted in four independent experiments for Neuro-2a cells (≥ 18 wells per experiment) and three independent experiments for HeLa cells (≥ 18 wells per experiment).

For proliferation assays, 8 × 10^3^ cells per well were seeded in 96-well plates, the plates were placed into an IncuCyte S3 live-cell imaging system, and images were acquired at 12-h intervals for up to 96 h. Proliferation was assessed by quantifying percent confluency with IncuCyte 2024B software. Proliferation assays were performed in five independent experiments for Neuro-2a cells (≥ 18 wells per experiment) and four independent experiments for HeLa cells (≥ 18 wells per experiment).

### RNA sequencing and gene ontology analysis

WT Neuro-2a cells and two independent Rab24 knockout clones (clone A and clone B) were used for RNA sequencing. Cells were seeded in 6-well plates and cultured to 80–90% confluency. Total RNA was extracted using NucleoSpin RNA extraction kit (Macherey–Nagel, 740955) according to the manufacturer’s instructions. Three biological replicates were prepared for each cell line. RNA samples were stored at − 80 °C and submitted to the Finnish Functional Genomics Centre (University of Turku and Åbo Akademi University) for RNA sequencing.

RNA quality was assessed by Fragment Analyzer (Advanced Analytical), the concentration was measured with Qubit Fluorometric Quantitation (Life Technologies). All samples passed the quality control criteria (RQN = 10). Library preparation was performed using Illumina TruSeq Stranded mRNA Library Prep Kit (Illumina, ligation protocol, 1000000124518) with 100 ng of input RNA. IDT for Illumina DNA/RNA UD Indexes were used for indexing. A human brain RNA sample (AM7962, Thermo Fisher Scientific) was included as a positive control. Average library fragment size was ~ 360 bp as determined by Fragment Analyzer. Sequencing was carried out on an Illumina NovaSeq 6000 SP platform with paired-end 2 × 50 bp read length, using a single pool over two lanes with a 1% PhiX spike-in. Each sample yielded 27–33 million reads. Base calling and automatic adapter trimming were performed using Illumina bcl2fastq2 software, and data were delivered as FASTQ files.

Downstream bioinformatic analyses were performed using standard pipelines. In brief, reads were quality-checked with FastQC and trimmed with Cutadapt. Clean reads were aligned to the mouse reference genome mm10 using a splice-aware aligner (STAR in two-pass mode to improve alignment accuracy). Protein-coding gene-level quantification was carried out with featureCounts, and differential gene expression analysis was performed using the statistical framework DESeq2 in R.

Gene ontology (GO) analysis (GOTERM_BP_DIRECT) was performed using DAVID (https://davidbioinformatics.nih.gov/summary.jsp, da Huang et al. 2009, Sherman et al. 2022). Significantly down- or upregulated genes with Log2 fold change smaller than –1 or higher than 1, respectively, were imported to DAVID. Minimum number of genes (count threshold) was set to 10.

### Analysis of RAB24 mRNA expression and patient survival using TCGA data

Publicly available RNA sequencing and patient survival data were retrieved from The Cancer Genome Atlas (TCGA) using the UALCAN portal (https://ualcan.path.uab.edu/; Chandrashekar et al. 2017, 2022). Six cancer types were selected for analysis, representing distinct RAB24 expression patterns observed in our IHC data: pancreatic adenocarcinoma (PAAD), HCC, lung squamous cell carcinoma (LUSC), sarcoma (SARC), glioblastoma multiforme (GBM), and kidney chromophobe carcinoma (KICH). For each cancer type, *RAB24* mRNA expression levels were compared between normal and tumour tissues using the TCGA level 3 RNA-seq data available through UALCAN. Box plots were generated directly by the portal, showing the 25th and 75th percentiles (boxes), median (line), and minimum and maximum values (whiskers). Overall survival analyses were performed through UALCAN using Kaplan–Meier estimates based on TCGA clinical data. Patients were divided into two groups according to *RAB24* expression level (high vs. low/medium), and statistical significance between survival curves was evaluated using the log-rank test, as implemented in the UALCAN tool.

### Statistics

Statistical analysis was performed with GraphPad Prism (GraphPad) using the tests indicated in the respective figure legends.

## Results

### Age-specific Rab24 levels in mouse tissues

**Fig. 1:**
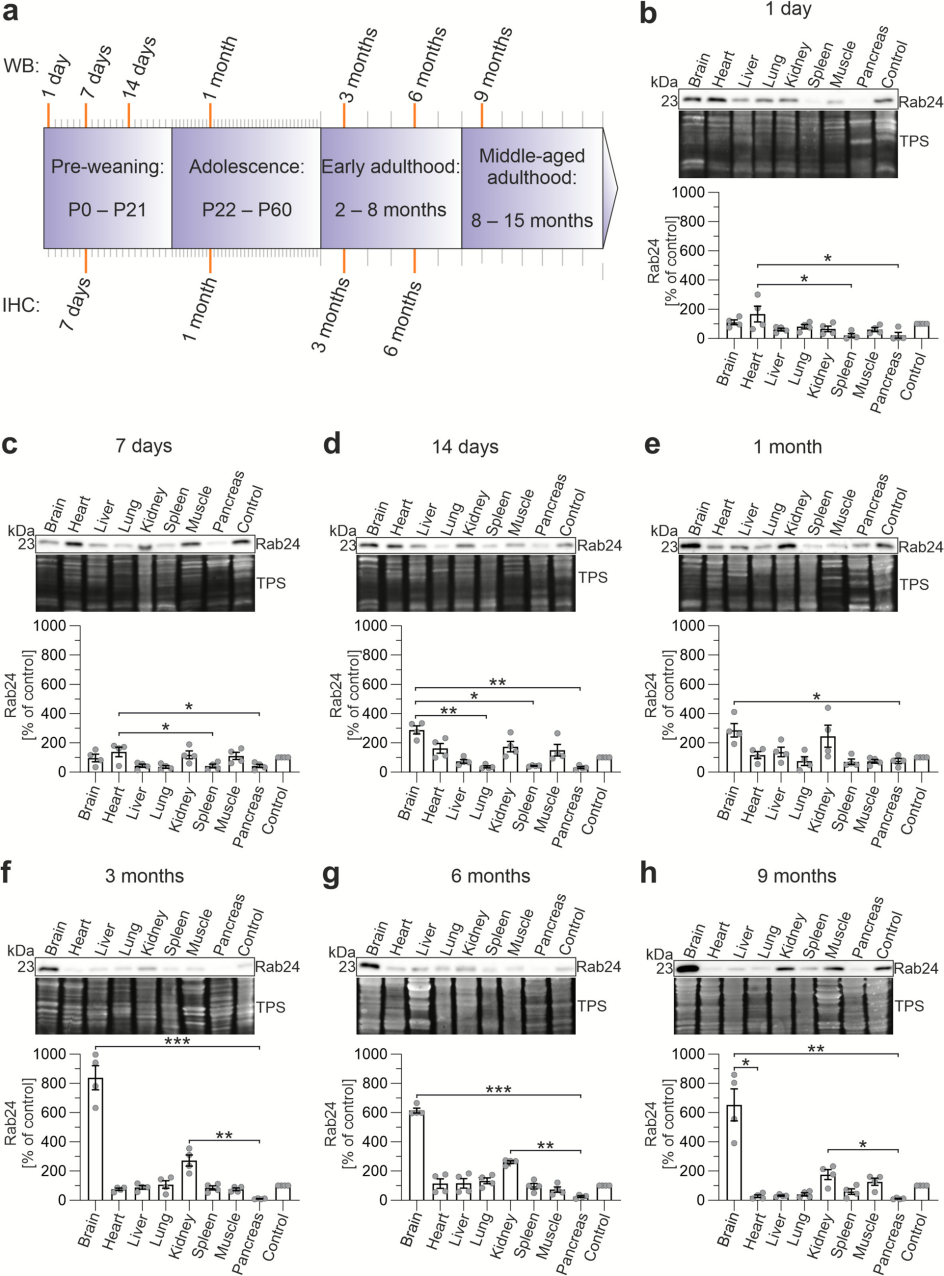
Rab24 protein levels vary in mouse tissues according to age. (**a**) Schematic drawing showing how the tissue samples were collected from seven age groups for western blotting (WB) and from four age groups for immunohistochemistry (IHC, indicated with orange bars). (**b**-**h**) Rab24 protein levels were analysed using western blotting with total protein staining (TPS) as the loading control. To compare Rab24 levels between different blots, tissue extract from one 1-month-old liver was used as a control sample in all blots. The Rab24 signals normalized to TPS were further normalized to the control in each blot. Representative western blot images, total protein staining, and quantification of Rab24 levels in the indicated organs at each age group are shown. The graphs show the mean and standard deviation calculated from four mice (two males and two females) in each age group. Kruskal–Wallis test and post hoc Dunn's multiple comparisons test were used for statistical significance: *p < 0.05, **p < 0.01, ***p < 0.001

Rab24 is involved in multiple cellular processes, including autophagy, endocytosis and cell division (Amaya et al. 2016; Militello et al. 2013; Yla-Anttila et al. 2015), making its developmental regulation particularly interesting. To compare Rab24 levels between organs at different ages, we analysed brain (cerebral cortex), heart, liver, lung, kidney, spleen, skeletal muscle (*tibialis anterior*), and pancreas samples by immunoblotting. The tissues were obtained from mice spanning from early postnatal age to middle-aged adulthood (1-day to 9-month-old, Fig. 1a). We utilized a total protein staining as a loading control for quantifications. Total protein staining has been shown to be reliable for normalizing the loading in tissues with varied proteomic profiles, as it reflects overall protein content and compensates for tissue-specific differences (Bettencourt et al. 2020, Musyaju et al. 2023).

First, we compared Rab24 levels across tissues within each age group, providing insight into organ-specific Rab24 levels. For comparative analysis between the different blots, the relative levels of Rab24 in each blot were normalized to a control sample, obtained from the liver of a 1-month-old mouse and loaded onto each gel (Fig. 1b-h). The liver was selected as the reference tissue due to its large size, which facilitates the preparation of ample, consistent cell extract. We did not observe any significant differences in Rab24 levels based on gender in any of the age groups (data not shown). In 1-day- and 7-day-old mice, Rab24 level varied between tissues, ranging from one fourth to twofold of the level in the liver control sample. The highest levels were observed in the heart in both age groups (Fig. 1b, c). A relatively high level was also observed in the brain at 1 day and 7 days, and in the kidney and skeletal muscle at 7 days. In contrast, the lowest Rab24 levels were detected in the spleen and pancreas at 1 day and 7 days, and in the liver and lung at 7 days (Fig. 1b, c).

In 14-day and 1-month-old mice, Rab24 levels increased, with particularly elevated levels in the brain followed by the kidney, heart and skeletal muscle at 14 days, and the kidney at 1 month (Fig. 1d, e). Conversely, the lung, spleen, and pancreas showed low Rab24 levels.

In 3-month-old mice, Rab24 levels of the brain showed a significant increase compared with the other tissues, which was consistent in the 6 and 9-month samples (Fig. 1f-h). In addition, Rab24 levels remained elevated in the kidney in the 3, 6, and 9-month samples (Fig. 1f-h).

Overall, these findings revealed dynamic changes in Rab24 levels across different tissues during postnatal development and aging, suggesting tissue-specific regulation of expression and possible tissue-specific functions for Rab24.

### Tissue-specific Rab24 levels according to age

To allow reliable comparisons of Rab24 levels within each organ during postnatal development and aging, we conducted additional western blots in which all samples from each tissue were loaded on the same gel. This allowed us to minimize gel-to-gel variability and to enhance the resolution of age-related changes within each tissue. In these comparisons, the Rab24 levels were normalized to the level observed in the 1-day-old sample in each tissue.

**Fig. 2:**
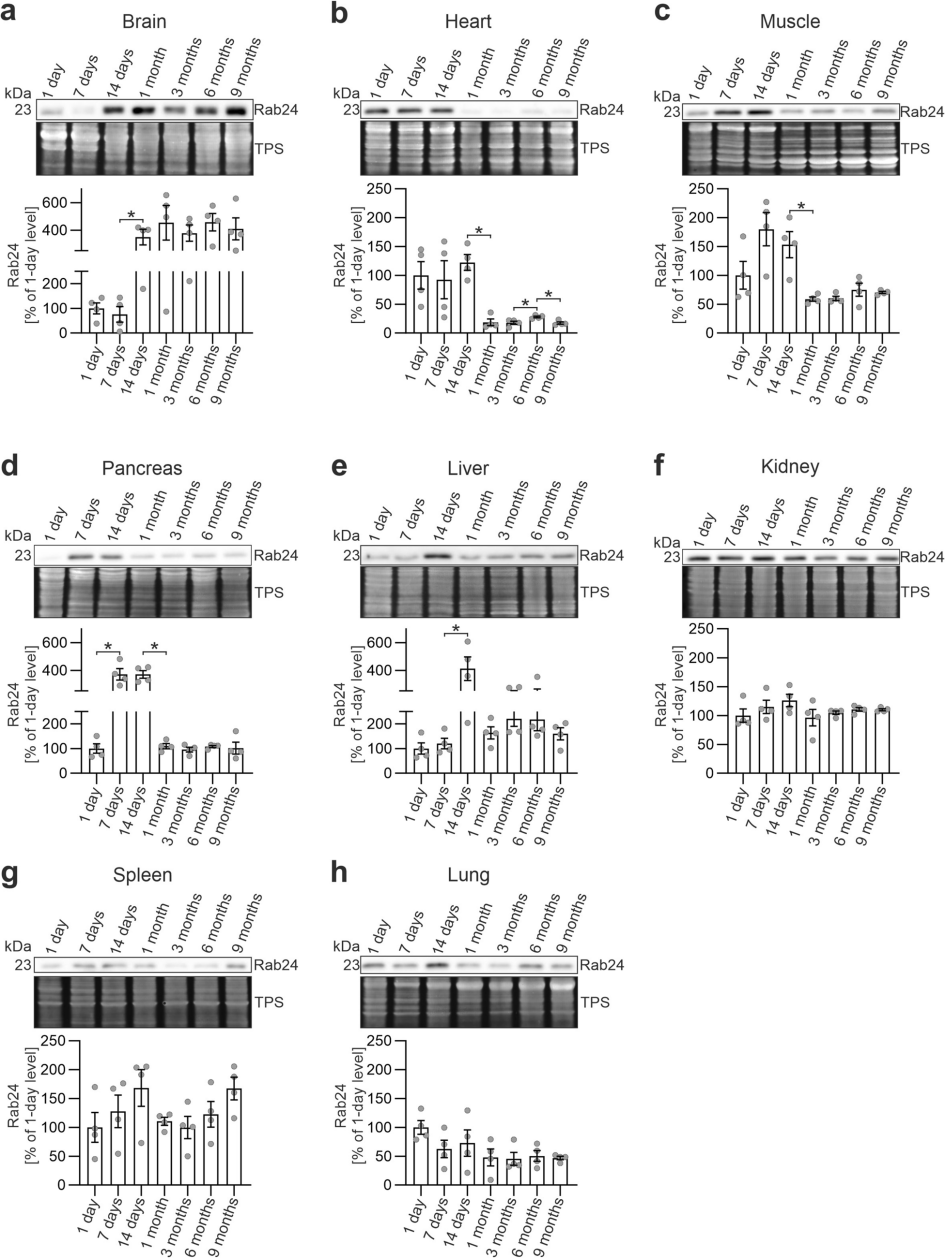
Rab24 protein levels in mouse tissues according to age. Rab24 protein levels were examined using western blotting with total protein staining (TPS) as the loading control. The Rab24 signals normalized to TPS were normalized to the 1-day old sample in each blot. (**a**-**h**) Representative western blot images, total protein staining, and quantification of Rab24 levels for each tissue are shown. The graphs show the mean and standard deviation calculated from four mice (two males and two females) for each age group. Mann–Whitney test was used for statistical significance of adjacent ages: *p < 0.05

Rab24 levels in the brain significantly increased starting at 14 days of age, with a 4–5-fold increase compared with the 1-day and 7-day-old samples (Fig. 2a). The elevated level was sustained in the 1-month, 3-month, 6-month, and 9-month-old brains.

In contrast to the brain, Rab24 levels in the heart and skeletal muscle peaked during early postnatal development (1–14 days), followed by a significant reduction in adult tissues (Fig. 2b, c). Rab24 levels in the pancreas and liver also displayed a notable pattern according to age. In both organs, Rab24 levels were initially lower in 1-day-old tissue, rose significantly by 7 days in the pancreas and by 14 days in the liver, then decreased at 1 month of age, and remained low in the 3- and 9-month samples (Fig. 2d, e).

In contrast to the dynamic changes seen in the tissues mentioned above, Rab24 levels in the kidney remained stable across all age groups (Fig. 2f). Rab24 levels in the spleen and lung also showed less variability according to age (Fig. 2g, h).

Collectively, these findings highlight distinct, age-dependent patterns of Rab24 protein across tissues, indicating the existence of tissue-specific regulatory mechanisms of Rab24 protein levels. In the brain, Rab24 levels increased after early development and remained elevated, suggesting a possible sustained role in postnatal housekeeping. Conversely, in the heart, muscle, pancreas, and liver, higher Rab24 levels were linked to early developmental stages, decreasing as the tissues matured. Finally, the kidney, spleen, and lung exhibited more stable Rab24 levels across all tested age groups.

### In the brain, Rab24 is predominantly localized to neurons in 1-month old and older mice

**Fig. 3:**
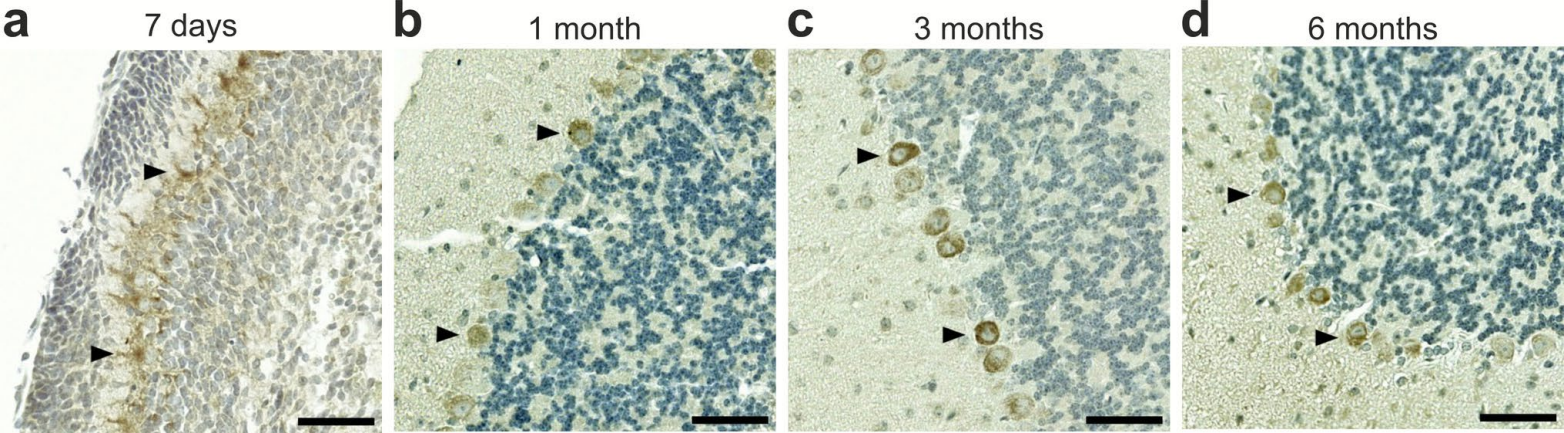
Rab24 immunohistochemical staining in the murine cerebellum according to age. (**a-d**) Representative images showing the cerebellum of mice aged 7 days to 6 months. Arrowheads indicate Rab24-positive Purkinje cells. The staining was performed on cerebellar sections from eight animals (four males and four females) per age group, representative images are shown. Scale bar: 50 µm

**Fig. 4:**
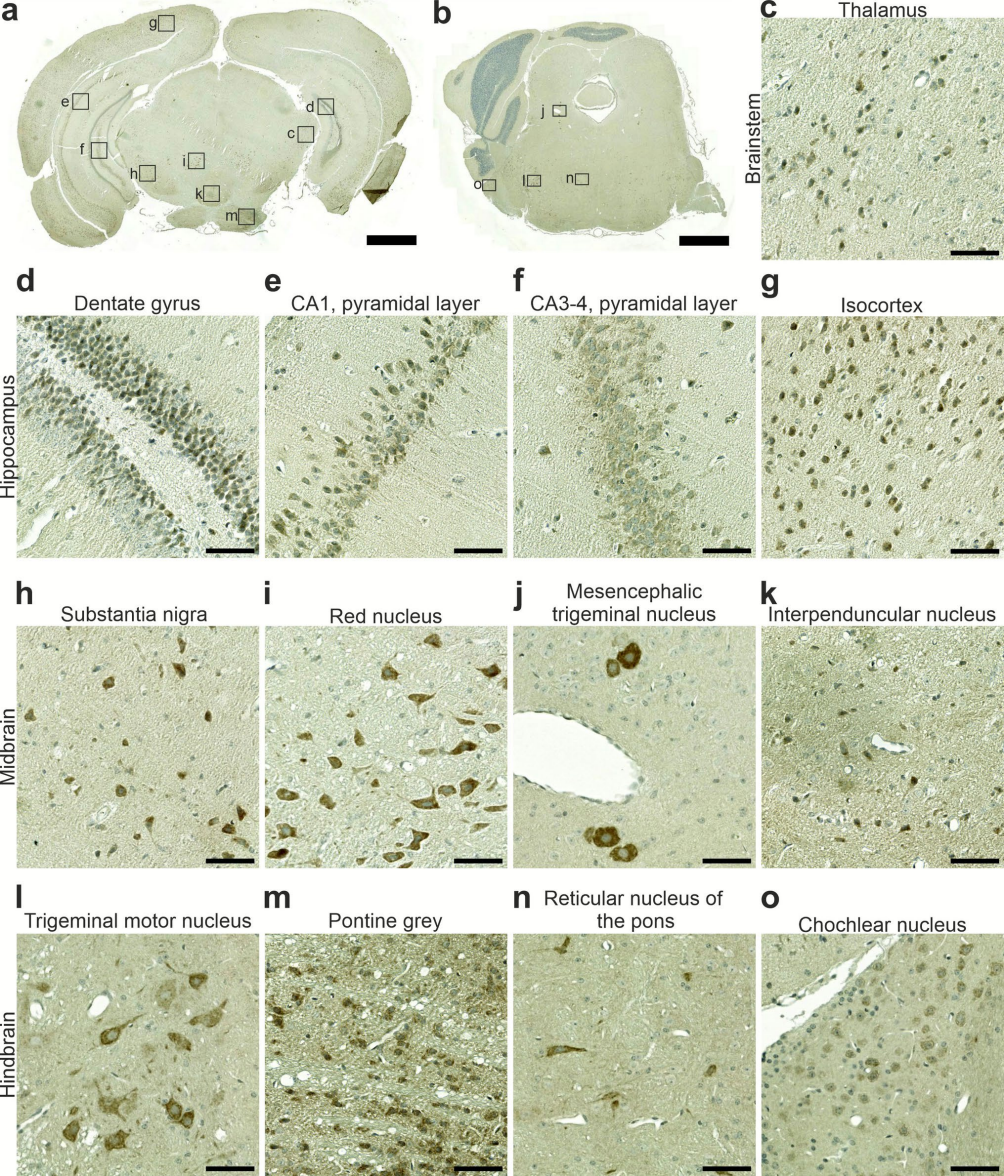
Rab24 immunohistochemical staining in 1-month-old mouse brain. (**a, b**) Low-magnification images showing the hippocampus, midbrain and hindbrain regions. The boxed areas in panels **a** and **b** indicate the regions shown at higher magnification in panels **c-o**. The brown Rab24-positive cells are neurons. The staining was performed on sections from eight animals (four males and four females); representative images are shown. Scale bar: 1 mm (**a**, **b**) and 50 μm (**c**-**o**)

Given that the brain exhibited the highest levels of Rab24 among all tested organs, we next investigated Rab24 localization across different brain regions and cell types using IHC. To explore potential changes in staining with age, we analysed brain tissue from mice aged 7 days, 1 month, 3 months, and 6 months (Fig. 1a). Based on the morphology of the Rab24-positive cells, the Rab24 protein was predominantly localized to neurons in various brain regions in 1-month-old and older brains. The staining patterns were consistent across biological replicates. Notably, Purkinje cells in the cerebellum exhibited Rab24 staining across all examined age groups, from postnatal day 7 to 6 months, with minimal age-related changes in staining intensity (Fig. 3a-d). In contrast, Rab24 staining was either absent or low in many other neuronal populations of 7-day-old mice (Fig. S4). In 1-month-old mice, increased Rab24 staining was observed in many neuronal populations across several brain regions (Fig. 4a-o). A consistent regional distribution of Rab24-positive neurons was observed in the thalamus, hippocampus, isocortex, midbrain, and hindbrain in 3- and 6- month-old mice (Figs S5 and S6). These findings are in agreement with the sustained high Rab24 protein levels after 14 days of age observed in western blot analyses of brain tissue (Fig. 2a).

Region-specific analysis revealed Rab24-positive neurons throughout the thalamus (shown for 1-month-old tissue in Fig. 4a, c). Within the hippocampus, weak to moderate Rab24 staining was detected in neurons of the dentate gyrus, as well as in the CA1 and CA3-CA4 subfields (Fig. 4a, d-f, S5a-c, S6a-c). In the isocortex, Rab24 staining was observed in neurons distributed across the entire cortical mantle (shown for 1-month-old tissue in Fig. 4a, g). In the midbrain, Rab24 was prominently stained in neurons of several sites, including the substantia nigra, red nucleus, mesencephalic trigeminal nucleus, and interpeduncular nucleus (Fig. 4a, b, h–k, S5d-f, S6d-f). Similarly, in the hindbrain, Rab24 staining was high in neurons of the trigeminal motor nucleus, pontine grey, and reticular nucleus of the pons (Fig. 4a, b, l-n, S5g-I, S6g-i), and low to moderate in neurons of the cochlear nucleus (shown for 1-month-old tissue in Fig. 4b, o).

Finally, we compared Rab24 staining in mouse brain with human adult brain tissue present in the central nervous tissue TMA CNS2081a (TissueArray.Com). Similar to mouse brain, neurons showed the strongest RAB24 staining in the adult human brain (Fig. S7a).

### Rab24 staining in different types of epithelial cells

**Fig. 5:**
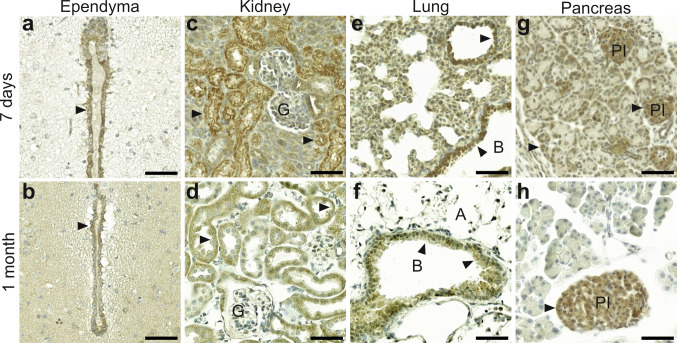
Rab24 immunohistochemical staining in epithelial cells in mouse tissues. Representative images showing Rab24 staining of 7-day-old (**a**, **c**, **e**, **g**) and 1-month old (**b**, **d**, **f**, **h**) mice. Ependyma lining the brain ventricles (**a**, **b**), kidney (**c**, **d**), lung (**e**, **f**), and pancreas (**g**, **h**) are shown. Arrowheads indicate Rab24-positive epithelial cells. G, kidney glomerulus; A, alveolus; B, bronchiole; PI, pancreatic islet. The staining was performed on sections from four animals (three males and one female) for 7-day-old mice and on sections from eight animals (four males and four females) for 1-month-old mice; representative images are shown. Scale bar: 50 µm

IHC revealed Rab24 staining in epithelial cells across multiple organs, with notable age-dependent differences in staining patterns. While 7-day-old mice exhibited somewhat varying epithelial staining patterns, tissues of older mice (1, 3 and 6 months) displayed more consistent staining intensities. Representative images from 7-day-old and 1-month-old mice show the age-related differences in epithelial Rab24 staining (Fig. 5a-h). These staining patterns were consistent across biological replicates.

Ependymal cells line the ventricles in the brain. In the ependymal layer, Rab24 staining was stronger compared to the surrounding brain tissue in 7-day-old mice (Fig. 5a). However, in older mice, Rab24 staining in the ependyma was more comparable to that of the adjacent tissue (shown for 1-month-old tissue in Fig. 5b).

In the kidney, Rab24 staining followed a similar pattern in younger and older animals, with higher staining intensity in the epithelial cells of the proximal convoluted tubules compared to the distal tubules and glomeruli (Fig. 5c, d). The less differentiated tubules showed weaker Rab24 staining in the 7-day-old kidney (Fig. 5c). In 1-month-old kidney, Rab24 staining was slightly stronger in the proximal tubules than in the distal tubules. Additionally, Rab24 staining was also observed in the parietal cells of the Bowman’s capsule (Fig. 5d).

In the lungs, Rab24 staining was prominent in the bronchiolar epithelium across all age groups, but differences were observed in the alveolar lining between young and old tissue. In 7-day-old mice, pneumocytes lining the alveolar walls showed stronger Rab24 staining (Fig. 5e) compared to older animals (shown for 1-month-old tissue in Fig. 5f). Despite this age-related decrease, the bronchiolar epithelial cells maintained consistently high Rab24 levels throughout all age groups.

In the exocrine pancreas, Rab24 staining was notably stronger in the acinar epithelial cells of 7-day-old mice (Fig. 5g) compared to older animals (shown for 1-month-old tissue in Fig. 5h). The endocrine cells of the islets of Langerhans consistently showed stronger Rab24 staining compared to the surrounding exocrine tissue across all age groups (Fig. 5g, h).

Finally, we compared Rab24 staining in mouse tissues with the corresponding human adult tissues that were present in the multicancer TMAs. Analysis of the human kidney, lung, and pancreas revealed RAB24 staining patterns similar to those observed in adult mouse tissues (Fig. S7b-e).

### Rab24 staining in the heart, skeletal muscle, liver and spleen

**Fig. 6:**
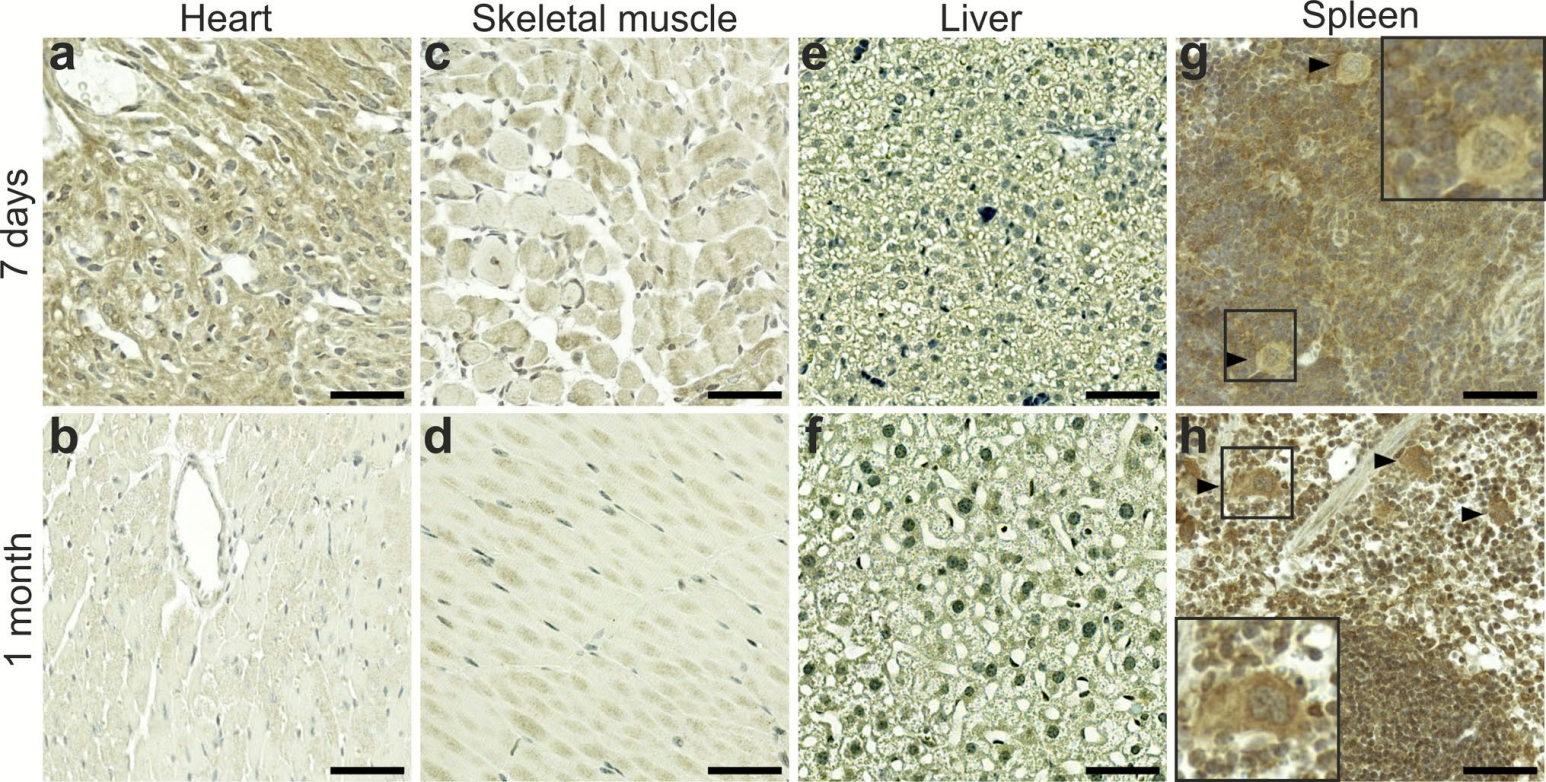
Age-dependent Rab24 immunohistochemical staining in the heart, skeletal muscle, liver, and spleen. Representative images showing 7-day-old (**a**, **c**, **e**, **g**) and 1-month old (**b**, **d**, **f**, **h**) tissues. (**g**, **h**) Insets and arrowheads indicate megakaryocytes in the spleen, showing low Rab24 staining at 7 days, and moderate Rab24 staining at 1 month. The staining was performed on sections from four animals (three males and one female) for 7-day-old mice and on sections from eight animals (four males and four females) for 1-month-old mice; representative images are shown. Scale bar: 50 µm

In the heart, Rab24 staining was moderate in cardiomyocytes of 7-day-old animals but markedly reduced in 1-month-old mice (Fig. 6a, b). In the older mice, both cardiomyocytes and cardiac endothelial cells showed low Rab24 staining (1-month-old tissue shown in Fig. 6b; similar staining was observed in 3- and 6-month-old tissue). A similar but less prominent age-dependent decrease in Rab24 staining was observed in the skeletal muscle (Fig. 6c, d). In contrast, Rab24 staining in the liver remained low in all age groups included in the IHC analysis (7-day-old and 1-month-old tissue shown in Fig. 6e and f; similar staining was observed in 3- and 6-month-old tissue). In the spleen, Rab24 staining was moderate in both 7-day-old and older animals, with uniform staining across the white and red pulp (Fig. 6g, h). In the spleen, megakaryocytes were weakly stained in 7-day-old tissue, and moderately stained in 1-month old and older tissue. These cells give rise to blood platelets.

Analysis of human adult tissues from the skeletal muscle and liver revealed RAB24 staining patterns similar to those observed in the adult mouse tissues (Fig. S7f, g).

### Comparison of Rab24 staining with markers of autophagic and endosomal/lysosomal compartments

Rab24 has functions in autophagy (Munafo and Colombo 2002; Yla-Anttila et al. 2015) and endosomal degradation (Amaya et al. 2016). To evaluate whether Rab24 staining patterns correlate with markers of these processes, we used sections from tissues showing age- or cell type-dependent changes in Rab24 staining (brain, kidney, lung, and pancreas). The sections were immunolabelled for marker proteins of autophagy (LC3, Beclin1, SQSTM1/p62) and for endosomal (Rab5, Rab7) and lysosomal (LAMP1 and LAMP2) compartments. LC3 localises to autophagosomal membranes, Beclin1 is part of phosphatidylinositol-3-kinase complexes that are needed for autophagosome formation and endosome maturation, and SQSTM1/p62 is an autophagy receptor that is degraded via autophagy. Rab5 and Rab7 associate with early and late endosomes, respectively, and LAMP1 and LAMP2 are lysosomal membrane proteins.

**Fig. 7:**
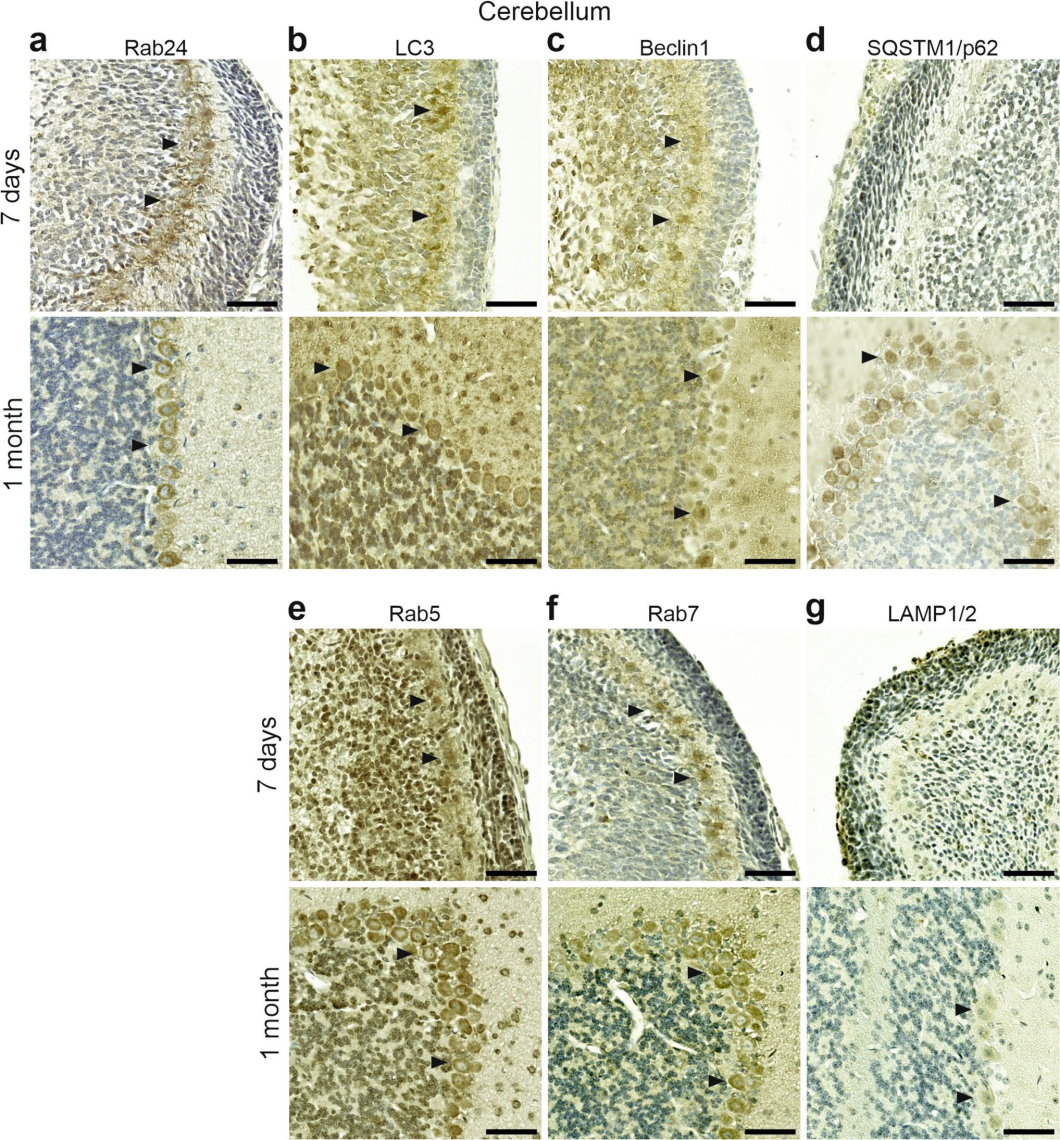
Immunohistochemical staining of Rab24 (**a**), autophagy markers LC3 (**b**), Beclin1 (**c**), and SQSTM1/p62 (**d**), endosomal markers Rab5 (**e**) and Rab7 (**f**), and lysosomal markers LAMP1 and LAMP2 (LAMP1/2) (**g**) in the cerebellum of mice aged 7 days and 1 month. Arrowheads indicate Purkinje cells positively stained for the respective marker. The staining was performed on sections from two animals for 7-day-old mice and two animals for 1-month-old mice; representative images are shown. Scale bar: 50 µm

In the cerebellum, Purkinje neurons displayed LC3 staining both at 7 days and 1 month (Fig. 7b) consistent with the Rab24 staining pattern (Figs. 3a, b and 7a). However, LC3 staining was also observed in the cells of the granular layer, especially at 1 month (Fig. 7b), unlike Rab24. Beclin1 showed weak to moderate staining in Purkinje cells and the granular layer at both 7 days and 1 month (Fig. 7c), while SQSTM1/p62 was negative at 7 days and positive in Purkinje cells at 1 month (Fig. 7d). Thus, LC3 and Beclin1 showed Rab24-like staining in Purkinje cells, but they were also positive in the granular layer unlike Rab24. SQSTM1/p62 was stained in Purkinje cells at 1 month like Rab24 but not at 7 days unlike Rab24.

Rab5 staining was observed in Purkinje cells and the granular layer at both 7 days and 1 month (Fig. 7e). Purkinje neurons stained positive for Rab7 at both time points, while overall staining was low in the surrounding tissue (Fig. 7f), resembling Rab24 distribution (Figs. 3a, b, 7a). LAMP1/2 staining was negative at 7 days and weakly positive in Purkinje cells at 1 month (Fig. 7g). Thus, among the endosomal-lysosomal markers, Rab7 staining pattern best resembled that of Rab24 in the cerebellum.

In the substantia nigra and thalamus, LC3-positive neurons were hardly distinguishable from the other cell types at 7 days but at 1 month, they were weakly or moderately stained and became visible (Figs S8b, S9b). Rab24 staining showed a similar pattern in neurons (Figs S8a, S9a) while the other cells types showed more staining for LC3 than Rab24 (Figs S8a, b, S9a, b). Beclin1 staining was observed in neurons at both 7 days and 1 month (Figs S8c, S9c), unlike Rab24. SQSTM1/p62 showed weak to moderate neuronal staining at both 7 days and 1 month in the substantia nigra (Fig. S8d) and no or weak staining in the thalamus (Fig. S9d). Thus, LC3 staining was similar to Rab24 in neurons but not in the other cell types in both age groups, Beclin1 staining resembled that of Rab24 at 1 month, and SQSTM1/p62 staining resembled Rab24 staining in both age groups in the thalamus and at 1 month in the substantia nigra.

In substantia nigra and thalamus, weak to moderate Rab5 staining was observed in neurons at 7 days and 1 month (Figs S8e, S9e). Neurons were not stained for Rab7 at 7 days but were positive at 1 month (Figs S8f, S9f), similar to Rab24. LAMP1/2 staining was absent or very weak in neurons (Figs S8g, S9g). Thus, in substantia nigra and thalamus, Rab7 and Rab24 showed a similar staining pattern, Rab5 staining was similar to Rab24 at 1 month, while LAMP1/2 staining showed limited correlation with that of Rab24.

To summarise, Rab7 and Rab24 showed similar staining patterns in the cerebellum, substantia nigra, and thalamus at both 7 days and 1 month. LC3 staining resembled Rab24 in Purkinje cells of the cerebellum and neurons of the substantia nigra and thalamus but not in other brain cell types. The remaining autophagy and endosomal-lysosomal markers resembled Rab24 staining pattern only partially in the brain tissue.

In the kidney, LC3 staining was present in the convoluted tubules and glomeruli at both 7-day and 1-month old tissue (Fig. S10b), whereas Rab24 was stained in the tubules but not in glomeruli (Figs. 5c, d, S10a). Beclin1 showed staining throughout the kidney at both 7-day and 1-month-old tissue (Fig. S10c). SQSTM1/p62 staining was weak overall at 7 days but became detectable in kidney tubules at 1 month (Fig. S10d), showing correlation with Rab24 staining at 1 month. Rab5 staining was detectable in glomeruli and part of tubules at 7 days and became restricted to tubules at 1 month (Fig. S10e), showing correlation with Rab24 staining in 1-month-old tissue. Rab7 staining was weak in kidney tubules and glomeruli at 7 days, but at 1 month it was detectable in the tubules while absent in glomeruli (Fig. S10f). Thus, Rab7 staining correlated with Rab24 in 1-month old kidney tubules. LAMP1/2 staining was strongly positive in part of tubules at both ages (Fig. S10g), resembling the pattern of Rab24 (Figs. 5c, d, S10a). In summary, LC3 and LAMP1/2 showed the best correlation with Rab24 staining in kidney tubules and glomeruli. In the adult kidney, all markers were detected in kidney tubular epithelium.

In the lung, LC3 staining was observed in the bronchiolar epithelium at both 7 days and 1 month, while the alveolar lining was positive at 7 days but negative at 1 month (Fig. S11b). Thus, LC3 staining showed correlation with Rab24 staining in the lung (Figs. 5e, f, S11a), especially at 1 month. Beclin1 staining shifted from high in alveolar lining to high in bronchiolar epithelium with age (Fig. S11c), showing correlation with Rab24 staining only at 1 month. SQSTM1/p62 staining was weak in the bronchiolar epithelium at both ages, and absent in alveolar cells at 1 month (Fig. S11d), showing some correlation with Rab24 staining in the alveolar lining (Fig. 5e, f, S11a). Rab5 staining was weak in both alveolar and bronchiolar cells at 7 days, becoming stronger and more restricted to bronchioles at 1 month (Fig. S11e). Thus, Rab5 staining was similar to Rab24 staining only at 1 month. Rab7 showed weak staining overall, with low positivity in the bronchiolar epithelium at both ages and in the alveolar lining only at 7 days (Fig. S11f). LAMP1/2 staining was observed in alveolar lining at 7 days and bronchiolar epithelium at 1 month (Fig. S11g), showing some correlation with Rab24 staining only at 1 month. To summarise, all markers were stained in bronchiolar epithelium at 1 month. Unlike Rab24, Beclin1, SQSTM1/p62, Rab5, Rab7 and LAMP1/2 showed weak or no staining in these cells at 7 days.

In the pancreas, LC3 staining was stronger in islet cells than in acinar cells at both 7 days and 1 month, with acinar staining higher at 7 days than at 1 month (Fig. S12b). Beclin1 (Fig. S12c) and SQSTM1/p62 (Fig. S12d) also showed stronger staining in the islets than in the acini, with acinar staining higher at 7 days. Thus, LC3, Beclin1 and SQSTM1/p62 staining patters resembled those of Rab24 (Figs. 5g, h, S12a). Rab5 staining was also strongly positive in the islets, with reduced acinar staining at 1 month (Fig. S12d). Rab7 staining displayed a similar Rab24-like pattern, with islets more strongly stained than acini, and acinar positivity decreasing from 7 days to 1 month (Fig. S12f). LAMP1/2 staining showed strong positivity in the islets, while acinar staining was weaker and decreased with age (Fig. S12g). In summary, markers of autophagy and endosomal/lysosomal compartments correlated most closely with Rab24 staining in the pancreas. Similar to Rab24, all markers showed strong staining in pancreatic islets at both age groups, while acinar staining was weaker and decreased with age.

### Rab24 knockout leads to downregulation of genes related to neuronal functions, cell signalling, and immunity

**Fig. 8:**
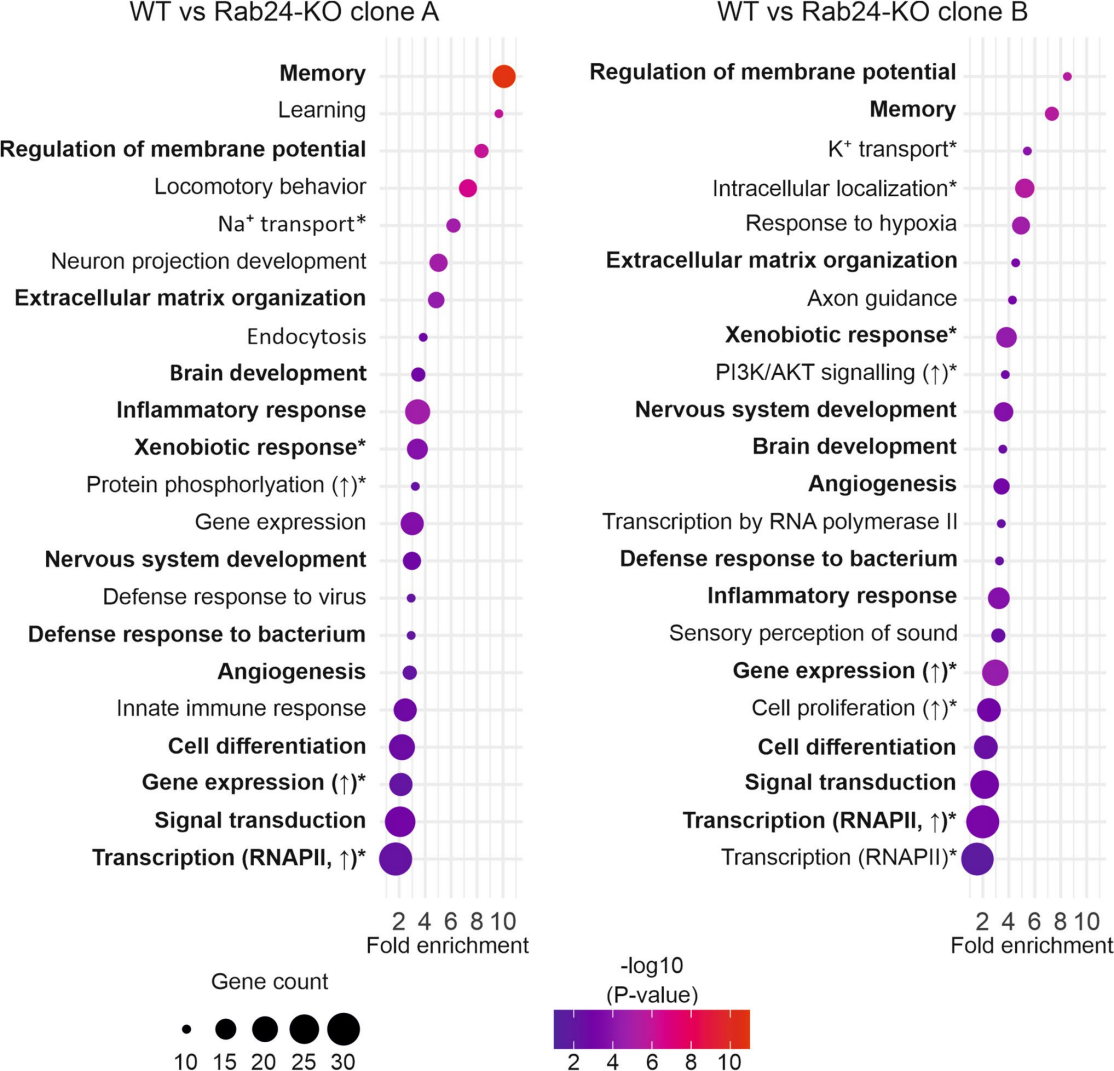
Gene Ontology (GO) enrichment analysis of genes downregulated in Rab24-deficient Neuro-2a cells in comparison with wild-type cells. In clone A, 421 genes were downregulated (left) and in clone B, 380 genes were downregulated (right). The GO terms represent biological processes, 22 most significant terms are shown for both clones. The bolded GO terms are common for clone A and clone B. In both panels, the x-axis shows the fold enrichment, which indicates how much more frequently a given GO term is represented among the downregulated genes than expected by chance. The size of each bubble corresponds to the gene count, i.e. the number of differentially expressed genes associated with that GO term. The colour scale represents the –log₁₀(p-value) of the enrichment test, reflecting the statistical significance of the enrichment (higher values indicate greater significance). For clarity, long GO term names were abbreviated in the plots and are marked with an asterisk (*): Na^+^ transport – Sodium ion transmembrane transport, Xenobiotic response – Response to xenobiotic stimulus, Protein phosphorylation (↑) – Positive regulation of protein phosphorylation, Gene expression (↑) – Positive regulation of gene expression, Transcription (RNAPII, ↑) – Positive regulation of transcription by RNA polymerase II, K^+^ transport – Potassium ion transmembrane transport, Intracellular localization – Establishment of localization in cell, PI3K/AKT signalling (↑) – Positive regulation of phosphatidylinositol 3-kinase/protein kinase B signal transduction, Cell proliferation (↑) – Positive regulation of cell population proliferation, Transcription (RNAPII) – Regulation of transcription by RNA polymerase II

To gain insight into Rab24’s cellular functions, we performed RNA sequencing of Rab24-KO and WT mouse Neuro-2a cells. We selected this model because Rab24 protein levels were highest in adult brain tissue (Fig. 1f-h), with the strongest staining in neurons (Fig. 4c-o). Two independent Rab24-KO clones were analysed alongside WT cells. Gene ontology (GO) analysis was performed for the genes differentially expressed in the Rab24-KO clones. To minimize the effect of clone-to-clone variability, we focused on GO terms that were among the 22 most significant for both Rab24-KO clones (Fig. 8). No common GO terms were identified for the genes upregulated in the two KO clones. On the contrary, for the genes downregulated in the Rab24-KO clones, thirteen GO terms were common for both KO clones (Fig. 8, bolded text). The GO terms *Memory*, *Regulation of membrane potential*, *Brain development*, *Nervous system development, Cell differentiation,* and *Angiogenesis* suggest possible roles for Rab24 in brain function and brain/nervous system development, which is in line with the high levels of Rab24 protein in the brain from postnatal day 14 onwards (Figs. 1 and 2), especially in neurons (Figs. 3 and 4).

Within the GO term *Extracellular matrix organization*, genes downregulated in both Rab24-KO clones included several collagen types and metallopeptidases. These suggest putative roles for Rab24 in cell adhesion, migration, and invasion. Cell adhesion and migration/invasion are important during development and cancer metastasis.

The immunity-related GO terms* Inflammatory response*, *Response to xenobiotic stimulus*, and *Defence response to bacterium *are in line with published results: Rab24 is known to localize to intracellular bacteria-containing vacuoles (Gutierrez et al. 2005) and Rab24 knockdown was reported to decrease antibacterial autophagy (Begun et al. 2012).

Under the GO term *Signal transduction*, genes downregulated in both Rab24-KO clones included brain-expressed X-linked 1, 2, and 3 (*Bex1, 2,* and *3*). Bex-family proteins function in signalling pathways regulating neuronal differentiation and are also implicated in cancers including glioma, liver cancer and breast cancer (Foltz et al. 2006, Wang et al. 2025). *Vgf* (nerve growth factor inducible) was also among the genes under the GO term *Signal transduction* in both Rab24-KO clones. Interestingly, in addition to functions in the brain (synaptic plasticity, neurogenesis, and neurite outgrowth (Thakker-Varia et al. 2014) and cancer, in particular brain metastasis (Carvalho et al. 2024), this gene product has been reported to protect pancreatic β-cells (Hirakida et al. 2023).

### Rab24 knockout decreased the migration and proliferation of Neuro-2a and HeLa cells

RAB24 promotes proliferation, migration, and invasion of HCC cell lines (Chen et al. 2017b). To investigate if Rab24 has a similar effect in other cell types, we tested two cell lines representing cell types that showed high Rab24 protein levels in our IHC analyses: Neuro-2a cells (neuroblastoma cell line, Rab24-KO clone A) and HeLa cells (originating from epithelial cells). Rab24-KO and WT cells were compared in scratch-wound and proliferation assays.

**Fig. 9:**
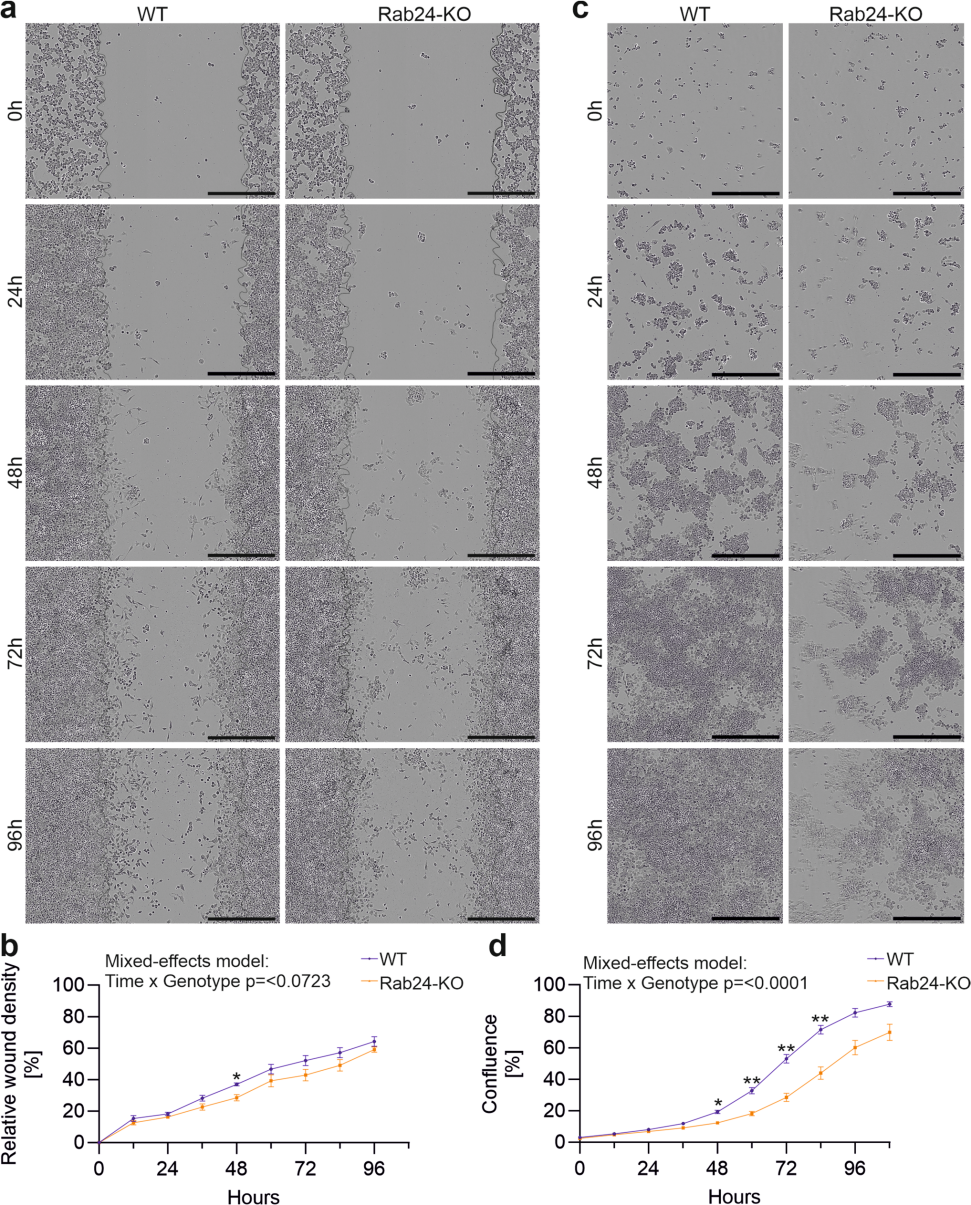
Rab24 knockout decreases migration and proliferation of Neuro-2a cells. (**a**) Representative images from scratch wound assays of wild type (WT) and Rab24 knockout (KO) Neuro-2a cells at the indicated time points. The thin black lines indicate the approximate location of the original scratch wound. (**b**) Quantification of relative wound density (%) in WT and KO cells over time. (**c**) Representative images from proliferation assays of WT and KO Neuro-2a cells at the indicated time points. (**d**) Quantification of cell confluence (%) in WT and KO cells over time. Points indicate the mean ± SEM. Each experiment was performed in n = 5 independent biological replicates, each with 12–24 technical replicates (wells) per genotype. Data were analysed using a mixed-effects model (REML) with Geisser–Greenhouse correction, including factors for Time and Genotype. Post-hoc Sidak’s multiple comparisons were performed between genotypes at each time point: *p < 0.05, **p < 0.01. Scale bar: 500 µM

In Neuro-2a cells, the scratch-wound assay revealed a slower wound closure in Rab24-KO compared to WT cells (Fig. 9a). Quantification of relative wound density showed a significant difference at 48 h, with WT cells displaying higher values than KO cells (Fig. 9b). However, the mixed-effects model did not reveal a significant time × genotype interaction for wound closure. In the proliferation assay, Rab24-KO cells expanded more slowly than WT cells (Fig. 9c), and quantification of confluence over time demonstrated significantly higher confluence in WT cells at 48, 60, 72, and 84 h (Fig. 9d). The mixed-effects model confirmed a significant time × genotype effect.

**Fig. 10:**
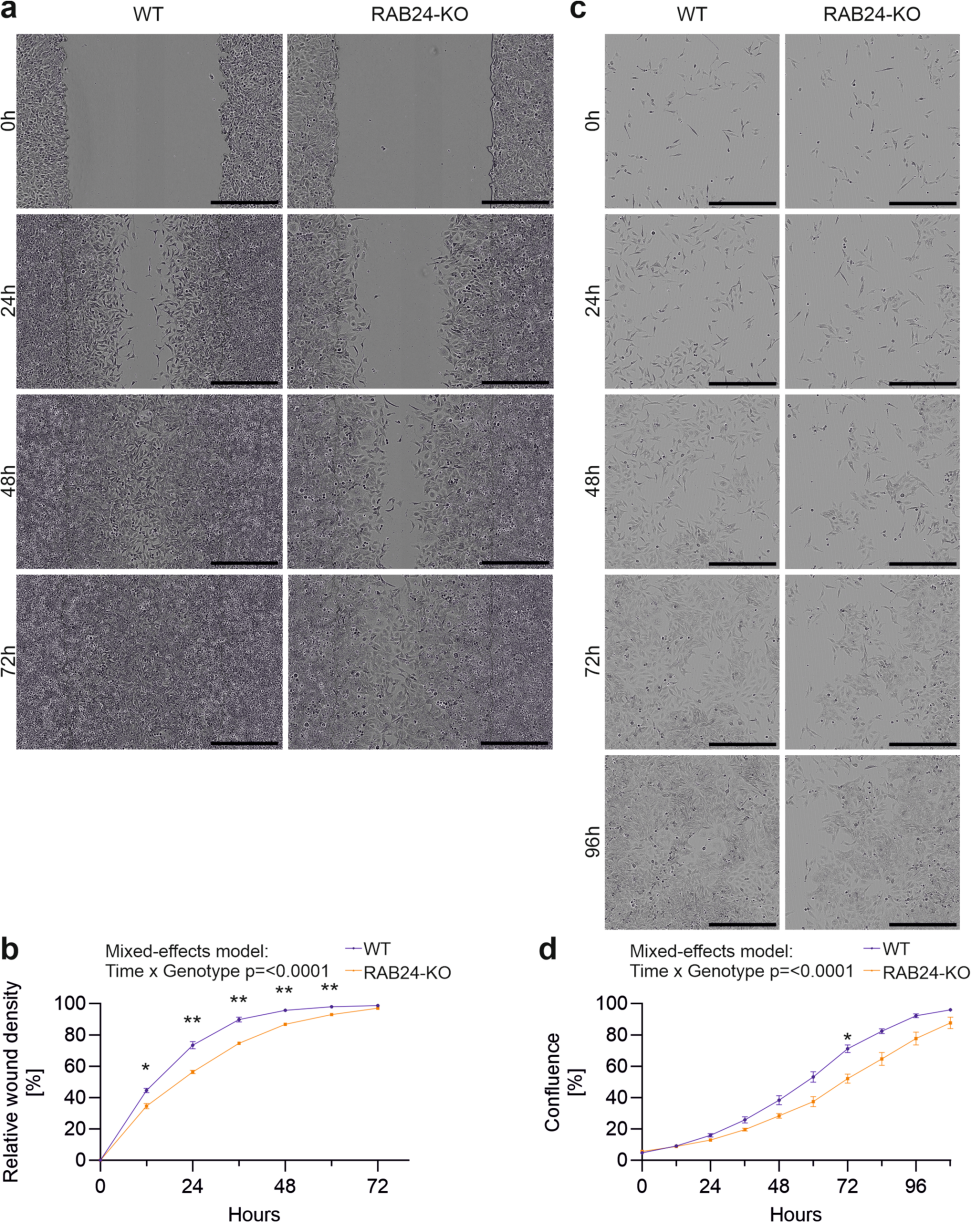
RAB24 knockout decreases migration and proliferation of HeLa cells. (**a**) Representative images from scratch wound assays of wild type (WT) and RAB24 knockout (KO) HeLa cells at the indicated time points. The thin black lines indicate the approximate location of the original scratch wound. (**b**) Quantification of relative wound density (%) in WT and KO cells over time. (**c**) Representative images from proliferation assays of WT and KO HeLa cells at the indicated time points. (**d**) Quantification of cell confluence (%) in WT and KO cells over time. Points indicate the mean ± SEM. Each experiment was performed in n = 5 independent biological replicates, each with 12–24 technical replicates (wells) per genotype. Data were analysed using a mixed-effects model (REML) with Geisser–Greenhouse correction, including factors for Time and Genotype. Post-hoc Sidak’s multiple comparisons were performed between genotypes at each time point: *p < 0.05, **p < 0.01. Scale bar: 500 µM

In HeLa cells, wound closure progressed more rapidly than in Neuro-2a cells, with RAB24-KO cells again showing delayed migration compared to WT cells (Fig. 10a). Quantification revealed significantly higher relative wound density in WT cells at 12, 24, 36, 48, and 60 h (Fig. 10b), and the mixed-effects model demonstrated a significant time × genotype effect. Similarly, in proliferation assays, RAB24-KO cells exhibited reduced confluence compared to WT cells (Fig. 10c). WT cells showed significantly higher confluence at 72 h (Fig. 10d), and the mixed-effects model confirmed a significant time × genotype effect.

In summary, Rab24 deficiency reduced migration and proliferation in both mouse Neuro-2a cells and human HeLa cells, in agreement with published results for human HCC cell lines (Chen et al. 2017b).

### RAB24 levels in human cancers

**Fig. 11:**
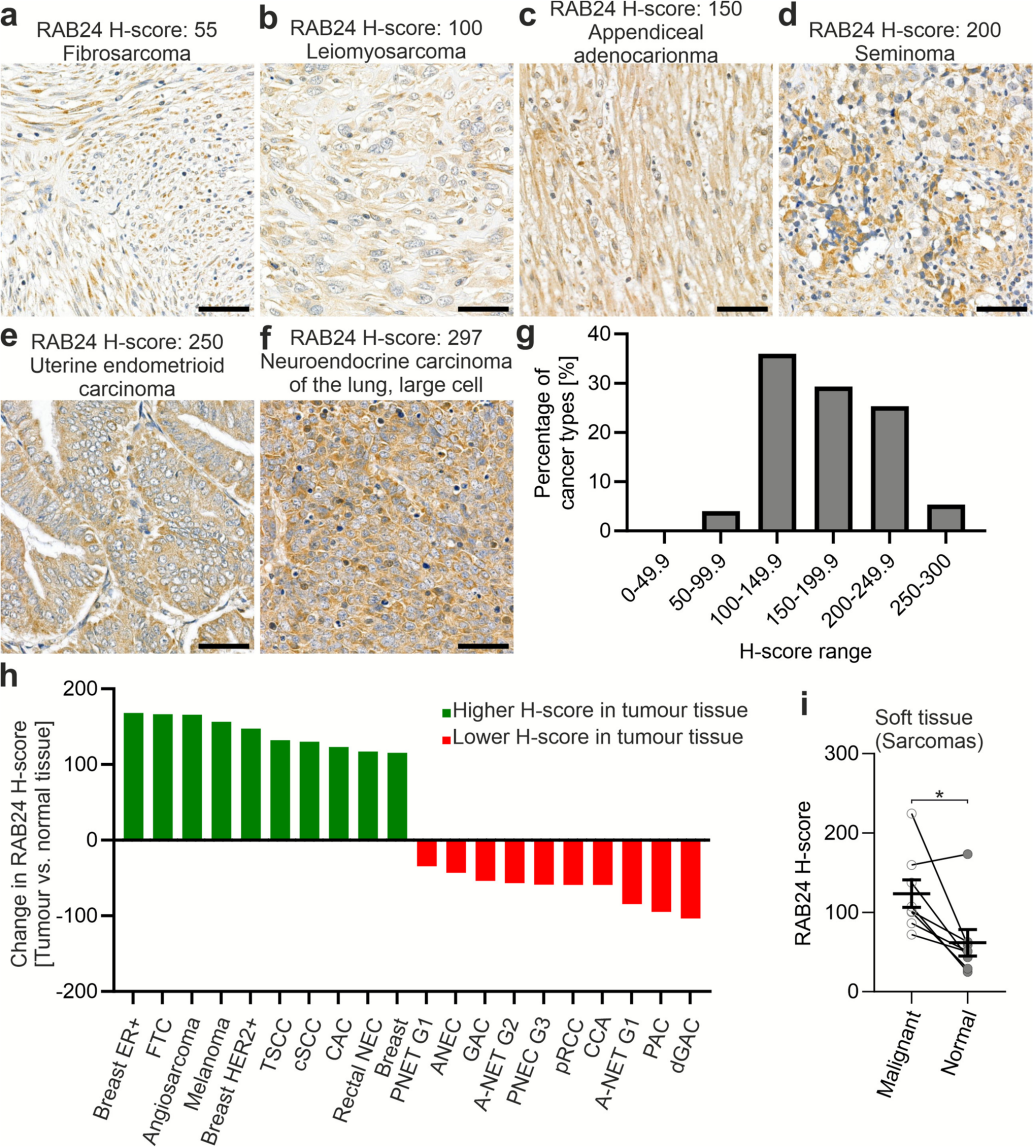
RAB24 immunohistochemical staining intensities across 75 cancer types analysed in multicancer tissue microarrays. The number of samples for each cancer type and the H-scores for RAB24 staining intensity are listed in Table S2. (**a**–**f**) Representative images showing RAB24 staining in malignant tissues exhibiting different RAB24 staining intensity and H-scores calculated using QuPath software. Scale bar: 50 µm. (**g**) Frequency distribution of RAB24 H-scores in the 75 cancer types. (**h**) Waterfall plot showing the top 10 cancer types with increased and decrease H-scores in malignant tissue compared with the corresponding normal tissue. Abbreviations: Breast ER +, ductal carcinoma of the breast, oestrogen-receptor positive; FTC, follicular carcinoma of the thyroid gland; Breast HER2 +, ductal carcinoma of the breast, human epidermal growth factor receptor 2-positive; TSCC, tonsillar squamous cell carcinoma; cSCC, cutaneous squamous cell carcinoma; CAC, cervical adenocarcinoma; Rectal NEC, rectal neuroendocrine carcinoma; Breast, ductal carcinoma of the breast, unspecified; PNET G1, pancreatic neuroendocrine tumour, low-grade (G1); ANEC, appendiceal neuroendocrine carcinoma; GAC, gastric adenocarcinoma; A-NET G2, appendiceal neuroendocrine tumour, intermediate grade (G2); PNEC G3, pancreatic neuroendocrine carcinoma G3; pRCC, papillary renal cell carcinoma; CCA, cholangiocarcinoma; A-NET G1, appendiceal neuroendocrine tumour, low-grade (G1); PAC, pancreatic adenocarcinoma; dGAC, diffuse gastric adenocarcinoma. (**i**) Paired dot plot of RAB24 levels in soft tissue cancers showing a statistically significant increase in RAB24 staining in malignant versus normal tissues. The horizontal lines indicate mean ± SEM. Statistical significance was determined by Wilcoxon test

RAB24’s known involvement in autophagy (Yla-Anttila et al. 2015), endocytosis (Amaya et al. 2016), mitosis (Militello et al. 2013), and cell proliferation and migration (Chen et al. 2017b, Figs. 9 and 10), processes frequently dysregulated in cancer, suggests that altered RAB24 protein levels may contribute to malignant transformation and tumour progression. Given the dynamic and tissue-specific levels of mouse Rab24 observed during postnatal development and aging, we sought to investigate whether RAB24 levels are altered in cancer. To address this, we first analysed RAB24 levels by IHC in 75 tumour types originating from 220 patients using multicancer TMAs (Table S2). RAB24 staining intensity was quantified using the H-scores (Fig. 11a-f), which were calculated with the QuPath software (Bankhead et al. 2017). The H-score is a semi-quantitative metric that combines staining intensity and the percentage of positively stained cells, providing a standardised method for comparing staining intensities in tissue samples. Frequency distribution of the H-scores revealed that RAB24 staining in the cancers included in the TMAs predominantly falls within the moderate range, with 65% of samples exhibiting H-scores between 100 and 199, while the maximum possible score is 300 (Fig. 11g). Strong RAB24 staining (H-score ≥ 200) was observed in 30.6% of the cancers, while low staining (H-score ≤ 99) was identified in the remaining 4.4% (Fig. 11g). In comparison, normal tissues removed alongside the tumours during surgery exhibited a more balanced distribution: 30.7% had lower RAB24 staining, 55.4% moderate staining, and 13.9% high staining (Fig. S13a).

To provide an overview, we summarized the findings for all 75 cancer types in a table that includes their tissue of origin, average RAB24 H-scores in malignant and normal tissues, the difference of H-scores between cancer tissue and normal tissue, and the number of patients per cancer (Table S2). The data reveal distinct patterns in RAB24 levels across cancers. The majority of the cancers demonstrated elevated RAB24 levels in malignant tissues compared to normal tissues, including several breast and skin cancer subtypes (Table S2, Fig. 11h). Conversely, 24% of cancers, particularly those of the digestive system (e.g., pancreas, stomach, appendix) and urinary tract, showed reduced RAB24 levels in malignant tissues compared with normal tissues (Table S2, Fig. 11h). A subset of cancers showed negligible changes in RAB24 levels (difference in H-score below 20) between malignant and normal tissues (Table S2).

For further analysis, the 75 cancer types were categorized into 21 groups based on tissue or organ of origin (Table S2, Fig. S1b). This categorization facilitated cross-comparison of RAB24 levels across diverse cancer types. For example, cancers of the thyroid, skin, and pancreas displayed high RAB24 staining, while cancers of the oesophagus, soft tissue, and stomach showed lower staining levels (Fig. S13b). Among the cancer samples in the TMA, only soft tissue sarcomas exhibited a statistically significant increase in RAB24 staining in malignant versus normal tissues (Fig. 11i). Other cancers demonstrated non-significant trends of increased RAB24 staining in malignant tissue (Fig. S13c-h), higher levels in normal tissues (Fig. S13i-k), or no substantial difference between normal and cancer tissue (Fig. S13l-n).

### RAB24 protein in brain cancers and neuroblastomas

Because we found neuronal localization of Rab24 in mouse brain, we next investigated whether RAB24 levels are altered in human neural tumours. Two TMAs were analysed, a central nervous system (CNS) tumour TMA including normal brain tissue and several brain tumour types (Table S4) and a neuroblastoma TMA including peripheral nerve and neuroblastoma subtypes (Table S5). RAB24 staining intensity was quantified using H-scores in QuPath.

**Fig. 12:**
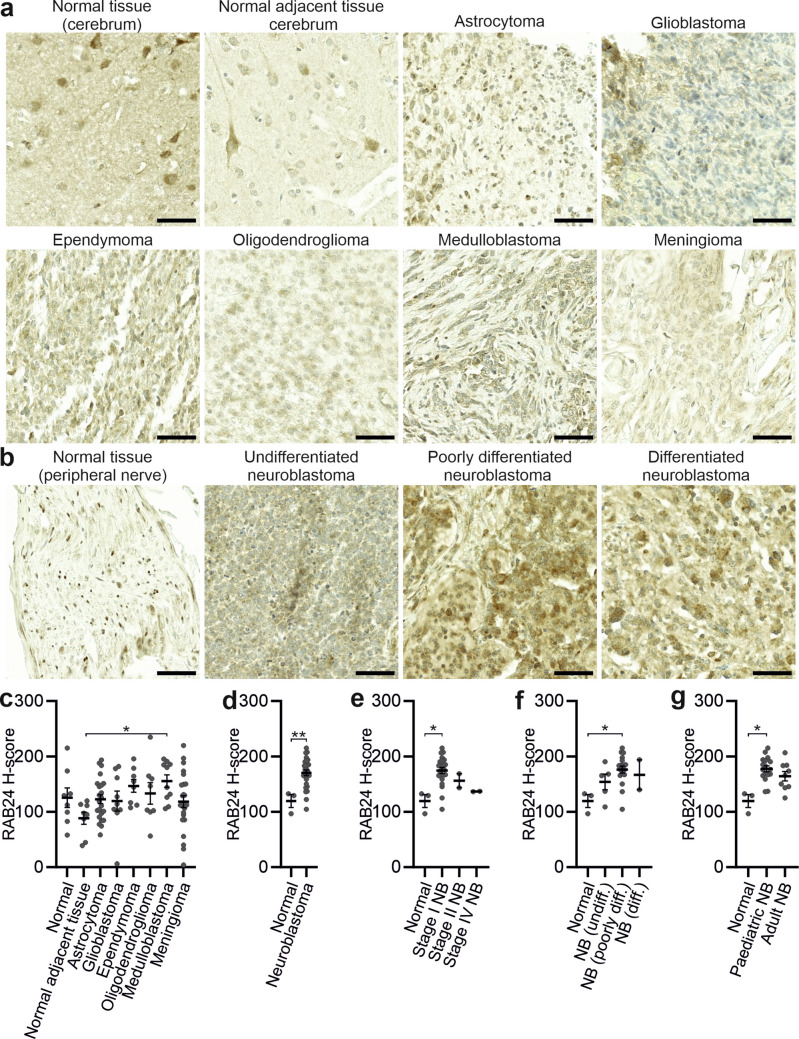
RAB24 immunohistochemical staining in central nervous system tumours and neuroblastomas. Tissue microarrays containing brain cancer or brain tissue samples from 104 patients (CNS2081a, Table S4) and neuroblastoma or peripheral nerve tissue samples from 30 patients (NB642d, Table S5) were from TissueArray.Com. (**a**) Representative images of RAB24 staining in tissues from the cerebrum and different tumours of the central nervous system. (**b**) Representative images of RAB24 staining in peripheral nerve, undifferentiated neuroblastoma, poorly differentiated neuroblastoma, and differentiated neuroblastoma. (**c**) Quantification of RAB24 H-scores across normal brain tissue, normal brain tissue adjacent to tumour, and brain cancers. (**d**) Quantification of RAB24 H-scores in normal peripheral nerve versus pooled neuroblastomas. (**e**) Quantification of RAB24 H-scores in neuroblastoma subtypes, classified according to International Neuroblastoma Staging System, compared to normal peripheral nerve. (**f**) Quantification of RAB24 H-scores in neuroblastoma differentiation subtypes compared to normal peripheral nerve. (**g**) Quantification of RAB24 H-scores in normal peripheral nerve compared to paediatric neuroblastoma (1–9 years of age) and adult neuroblastoma (18–84 years of age). The number of samples and the RAB24 H-scores in the TMAs are listed in Tables S4 and S5. Statistical significance was determined by Kruskal–Wallis test with Dunn’s post hoc correction (**c**, **e**, **f**, **g**) and Mann–Whitney test (**d**): *p < 0.05, **p < 0.01. Scale bar: 50 µM

In the CNS TMA, RAB24 staining was observed in normal cerebrum as well as across all tumour types with variable intensity (Table S4, Fig. 12a, c). The TMA included both normal and normal-adjacent tissue samples that were not anatomically matched to the tumour samples. Because Rab24 protein levels vary regionally within the brain (Figs. 4, S5, S6), this variability may contribute to the difference in H-scores between normal and normal adjacent tissue observed among the normal brain samples (Fig. 12a, c). Compared to adjacent normal brain tissue, a significant increase in RAB24 H-score was detected in medulloblastoma (Fig. 12c), a common paediatric cancer originating from neuronal progenitor cells (Sheng et al. 2024). There were no significant differences in the H-scores between normal brain and astrocytoma, glioblastoma, ependymoma, oligodendroglioma, and meningioma, even though there was a trend of increased RAB24 staining in the tumours (Fig. 12c). No differences in RAB24 H-scores were observed when comparing normal to inflamed brain tissue (Fig. S14a) or paediatric (up to 14 years of age) versus adult CNS tumours (Fig. S14b). We also compared RAB24 H-scores between different cancer grades in astrocytoma (Fig. S14c), oligodendroglioma (Fig. S14d), and meningioma (Fig. S14e), and observed no grade-dependent variation.

Neuroblastoma is the most frequently-occurring extracranial tumour in children, originating from neural crest cells (Alkhazal et al. 2025). In the neuroblastoma TMA (Table S5), RAB24 staining was consistently stronger in tumour tissue compared to normal peripheral nerve (Fig. 12b, d). When comparing tumours classified according to the International Neuroblastoma Staging System (INSS), the highest and significantly elevated RAB24 H-scores were detected in Stage I neuroblastomas (Fig. 12e). Subtype analysis revealed a significant increase in RAB24 H-scores in poorly differentiated neuroblastomas relative to normal tissue, while differences between normal tissue and undifferentiated and differentiated neuroblastomas were not significant (Fig. 12b, f). Stratification by patient age revealed higher RAB24 H-scores in paediatric neuroblastomas compared to normal tissue, while the difference between adult neuroblastomas and normal tissue did not reach significance (Fig. 12g). No significant differences in RAB24 H-scores were detected among neuroblastomas originating from different anatomical sites (adrenal gland, mediastinum, pelvic cavity, retroperitoneum) and normal peripheral nerve tissues (Fig. S14f).

In summary, elevated RAB24 H-scores were observed in medulloblastoma compared to normal adjacent brain tissue as well as in neuroblastoma compared to normal peripheral nervous tissue. Brain tumours of non-neuronal origin did not show significantly different RAB24 staining compared with normal brain tissue.

### RAB24 levels in pancreatic neuroendocrine tumours (PNETs)

**Fig. 13:**
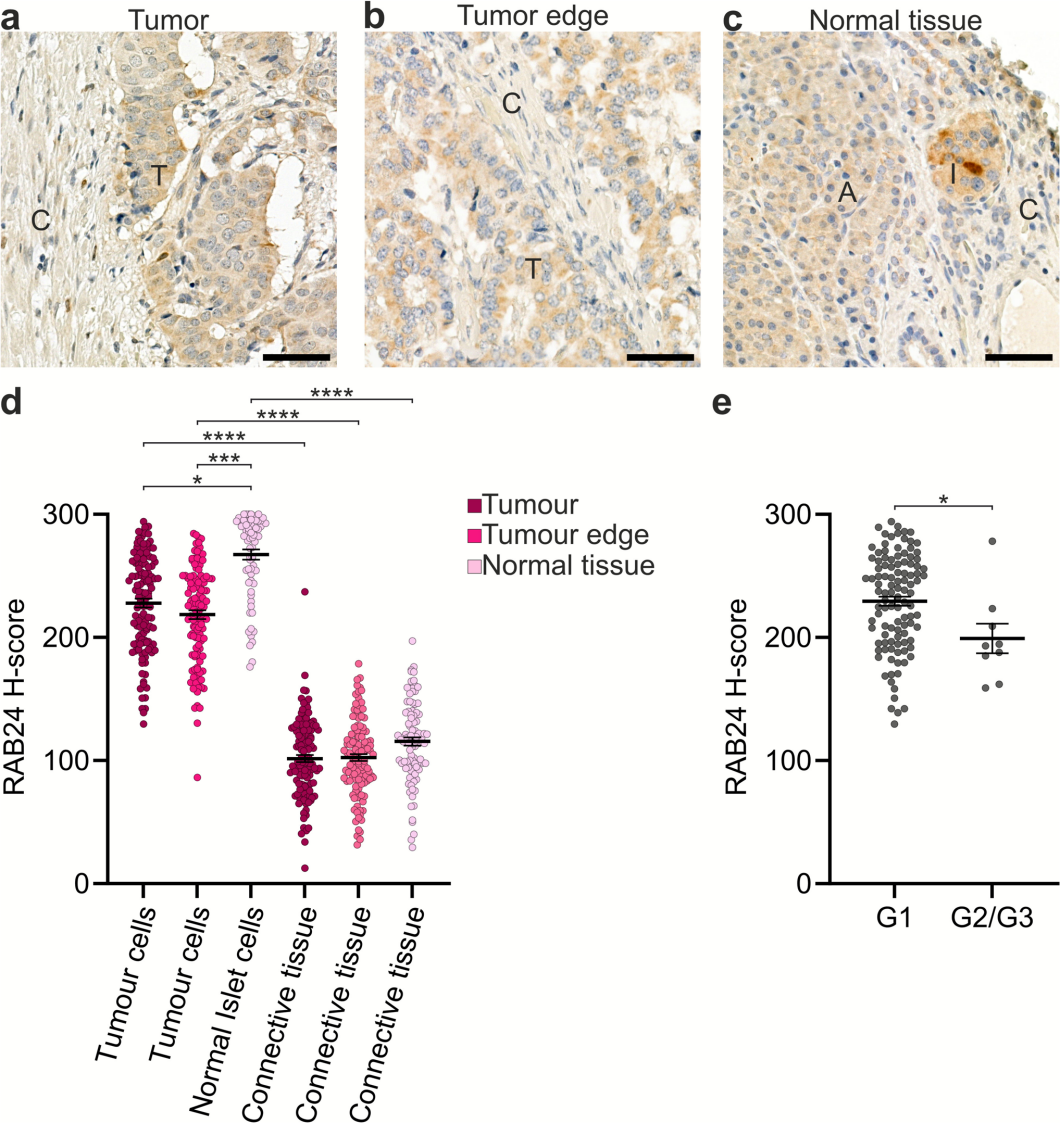
RAB24 immunohistochemical staining in pancreatic neuroendocrine tumours (PNETs) and normal pancreatic islets analysed in tissue microarrays. (**a**–**c**) Representative images of RAB24 staining in (**a**) tumour tissue, (**b**) tumour edge, and (**c**) normal pancreatic tissue. The tissues in the representative images show H-scores close to the mean H-score of the respective tissue type. Connective tissue (C), tumour (T), acinar tissue (A), and pancreatic islet (I) are indicated. Scale bar: 50 µm. (**d**, **e**) Quantification of RAB24 H-scores. H-scores were determined using QuPath software for tumour cells, islet cells, and connective tissue (**d**). RAB24 H-scores in low-grade (G1) PNET samples were significantly higher than in the intermediate (G2) and high-grade (G3) PNET samples (**e**). The number of samples and the RAB24 H-scores are listed in Table S3. Individual data points and mean ± SEM are shown, with statistical significance determined by Kruskal–Wallis test and post-hoc Dunn’s multiple comparison test (**d**), and Mann–Whitney test I (**e**)

RAB24 expression was reported to be decreased in patient samples and cell lines of PAAD, which originates from the pancreatic exocrine acinar cells. RAB24 was also identified as an independent low-risk factor in this cancer type (Deng et al. 2022, Yu et al. 2021). However, there are no data on RAB24 expression in PNETs, which originate from the endocrine pancreatic islet cells. Therefore, we analysed RAB24 protein levels in PNET TMAs containing samples from 120 patients (Table S3). The TMAs contained tissue samples from three regions: the tumour, the tumour edge, and normal pancreatic tissue (Fig. 13a-c). IHC staining for RAB24 was performed, and the staining intensity was analysed using H-scores. In tumour and tumour edge samples, the H-score was calculated separately for tumour cells and connective tissue, while in normal tissue samples, the H-score was determined for islet cells (the tissue of origin for PNETs) and connective tissue.

The analysis revealed significantly higher RAB24 staining in pancreatic islet cells from normal tissue compared to the tumour and tumour edge samples (Fig. 13d). No difference in RAB24 levels was observed between tumour and tumour edge samples. Furthermore, in connective tissue, RAB24 staining was significantly lower than in tumour cells. In normal tissue, RAB24 staining was lower in connective tissue compared to islet cells (Fig. 13d). Notably, no differences in RAB24 levels were detected between connective tissue in tumour, tumour edge, and normal tissue (Fig. 13d).

To determine whether RAB24 protein levels vary with tumour grade, we performed IHC staining for Ki-67, a well-established cell proliferation marker (Fig. S15). Based on Ki-67 indices (Nagtegaal et al. 2020), the majority of PNET samples (92.2%) were classified as low-grade (G1, 0–3% Ki-67-positive cells), while 6% were classified as intermediate-grade (G2, 3–20% Ki-67-positive cells), and 1.7% as high-grade (G3, more than 20% Ki-67-positive cells). Quantitative analysis of RAB24 H-scores revealed a significant decrease in RAB24 staining in higher-grade tumours, with PNET G1 samples displaying higher RAB24 levels compared to G2 and G3 tumours (Fig. 13e).

### RAB24 protein and mRNA levels correlate with patient survival in some cancers

To explore the possible clinical relevance of RAB24 in cancer, we compared our IHC results with *RAB24* mRNA expression and patient survival data in The Cancer Genome Atlas (TCGA) using the UALCAN portal (Chandrashekar et al. 2017, 2022).

Six cancer types were analysed, representing distinct *RAB24* expression patterns: PAAD, (Fig. S16a), HCC (Fig. S16b), lung squamous cell carcinoma (Fig. S16c), sarcoma (Fig. S16d), glioblastoma multiforme (Fig S16e) and kidney chromophobe carcinoma (Fig. S16f).

In PAAD, *RAB24* mRNA levels were significantly lower in tumours than in normal pancreas (Fig. S16a), consistent with our multi-cancer TMA IHC results (Table S2). Moreover, patients with high *RAB24* expression showed significantly improved overall survival (Fig. S16a), in agreement with published results (Deng et al. 2022, Yu et al. 2021). In contrast, *RAB24* mRNA was significantly upregulated in HCC relative to normal liver (Fig. S16b), consistent with our IHC findings showing higher H-scores in malignant hepatocytes (Table S2). Patients with high *RAB24* mRNA expression in tumours had reduced overall survival (Fig. S16b), in agreement with published results linking RAB24 overexpression to aggressive HCC and shorter patient survival (Chen et al. 2017b; Gu et al. 2025; He et al. 2002; Yang et al. 2020; Zhang et al. 2020; Zhu et al. 2020). In lung squamous cell carcinoma, neither *RAB24* mRNA expression (Fig. S16c) nor protein levels in our multicancer TMAs (Table S2) differed between normal and tumour tissue, and no significant differences in overall survival were observed between high and low/medium *RAB24* expression groups (Fig. S16c). In sarcoma, *RAB24* mRNA levels were elevated in tumour tissue compared to normal tissue (Fig. S16d). This pattern paralleled our multicancer TMA data, where the analysed sarcoma subtypes showed higher H-scores in malignant compared to benign tissues (Table S2, Fig. 11i). Higher *RAB24* expression was associated with better overall survival in sarcoma patients (Fig. S16d). In glioblastoma multiforme, *RAB24* mRNA levels were significantly lower in tumour samples than in normal brain tissue (Fig. S16e). Survival analysis indicated poorer prognosis in patients with high *RAB24* expression (Fig. S16e). These findings differ from our CNS TMA results, where glioblastomas showed RAB24 protein levels similar to adjacent normal tissue (Fig. 12c). This may be due to different patient cohorts in the two data sets, or it may reflect the fact that mRNA and protein levels do not necessarily match. Finally, in kidney chromophobe carcinoma, *RAB24* mRNA expression was higher in normal tissue than in tumours (Fig. S16f), consistent with our TMA results showing lower RAB24 H-scores in malignant compared to benign kidney tissue (Table S2). However, overall survival did not differ significantly between high and low *RAB24* expression groups (Fig. S16f).

These examples show that RAB24 mRNA and protein levels may show favourable or unfavourable correlation with patient prognosis, or no correlation at all, depending on the cancer type.

## Discussion

Our study provides the first comprehensive, cell-type-resolved analysis of Rab24 protein levels in mouse tissues from early postnatal age to adulthood. This approach extends beyond existing transcriptomic resources such as GTEx and the Human Protein Atlas, which report mRNA or protein levels primarily in adult tissues and lack cell-type-specific and dynamic profiling of protein levels across developmental stages. By combining western blotting and IHC, we reveal distinct tissue- and cell-type-specific patterns of Rab24 protein expression and dynamic changes during postnatal development. Importantly, we wish to point out that Rab24 protein abundance does not necessarily indicate functional significance; however, our data provide a foundation for future mechanistic studies addressing the tissue- and cell-type-specific functions of Rab24. Moreover, by analysing human cancers, we observed disease-specific alterations in RAB24 protein levels compared with peritumoral or normal tissues, suggesting that developmental and pathological regulation may be linked.

### Age- and tissue-specific changes in Rab24 protein level in mouse tissues

Rab24 levels in several tissues showed dynamic changes during early postnatal development, followed by stabilization in adulthood. These findings indicate that Rab24 may contribute to cellular processes during postnatal organ development, with a potential role in tissue homeostasis in maturity. However, as pointed out above, protein levels may not necessarily reflect functional roles. While our data on the protein levels are descriptive, previous studies support functional relevance for Rab24 in neuronal maintenance (Agler et al. 2014; Schwarz et al. 2025) and liver function (Seitz et al. 2019).

In the brain, Rab24 levels increased significantly between postnatal days 7 and 14 and remained elevated into adulthood. IHC showed that while Purkinje cells showed Rab24 staining in all age groups, other types of neurons had the highest Rab24 levels in 1-month-old and older brain. The increase in brain Rab24 level between 7 and 14 days coincides with the peak of synaptogenesis, a period marked by heightened neuronal activity and metabolic demands (Faria-Pereira and Morais 2022; Li et al. 2010). Consistent with reports showing that Rab24 localizes to synaptic vesicles (Taoufiq et al. 2020), our RNA sequencing analysis of Rab24-deficient Neuro-2a cells revealed differential expression of genes involved in memory, regulation of membrane potential, brain and nervous system development, cell differentiation, signal transduction, and transcription, supporting possible roles for Rab24 in neuronal development and function. In addition to synaptogenesis, other key processes are also activated in the mouse brain during postnatal days 1–10, including production, migration and differentiation of additional neurons, and selective elimination of unnecessary neurons by programmed cell death (Chen et al. 2017a). In line with this, our results showed that Rab24 supported the proliferation and migration of Neuro-2a cells. These findings collectively support the idea that Rab24 contributes to brain development, neuronal maturation and long-term neuronal maintenance.

Within the brain, immunohistochemistry confirmed robust Rab24 levels in different types of neurons, including neurons involved in sensory processing, motor control, and coordination, such as Purkinje cells. Interestingly, Rab24 protein staining in Purkinje cells was already evident at 7 days of age and stayed similar until adulthood. This aligns with prior evidence showing that a Rab24 mutation leads to Purkinje cell degeneration in dogs (Agler et al. 2014), underscoring the essential role of Rab24 in maintaining this specialized neuronal population.

Similar to Rab24, the autophagy markers LC3 and Beclin1 and the endosomal markers Rab5 and Rab7 showed immunostaining in Purkinje cells in both age groups tested. Rab7 staining was similar to Rab24 in neurons of the cerebellum, substantia nigra and thalamus and LC3 staining resembled that of Rab24 in neurons of the substantia nigra and thalamus. These findings suggest that neurons in these brain regions are active in autophagy and endocytosis. Rab24 has confirmed roles in both processes (Amaya et al. 2016; Munafo and Colombo 2002; Yla-Anttila et al. 2015).

In several peripheral tissues, including the heart, skeletal muscle, pancreas, and liver, Rab24 levels peaked during the first two postnatal weeks and declined thereafter. The elevated Rab24 levels coincide with a developmental period characterized by increased biosynthetic and metabolic activity associated with organ maturation. In the heart and skeletal muscle, Rab24 levels peaked between 1 and 14 days of age, a period of rapid cellular growth and differentiation (Gattazzo et al. 2020; Li et al. 1996), before decreasing to stable levels in adulthood. The initially higher levels of Rab24 in the heart coincide with a period of rapid growth and cellular differentiation of cardiomyocytes (Piquereau et al. 2010). Similarly, Rab24 level in the liver and pancreas was notably elevated at 7 and 14 days (pancreas) or 14 days (liver) of age compared to older tissues, correlating with the high metabolic and biosynthetic demands of these tissues during postnatal development (Bonner-Weir et al. 2016; Liang et al. 2022).

Notably, Rab24 level in the pancreatic islets of Langerhans remained consistently higher than in the surrounding exocrine tissue in all age groups, suggesting a possible role for Rab24 in these hormone-secreting cells. Interestingly, the strongest correlation between Rab24 and multiple markers of autophagy and lysosomal compartments was observed in the pancreatic islets. IHC staining for LC3, Beclin1, SQSTM1/p62, Rab5, Rab7, and LAMP1/2 revealed strong signals in the islet cells, suggesting high autophagic and endosomal/lysosomal activity in this cell population. These findings support the hypothesis that Rab24 may contribute to lysosome-related processes in the pancreatic endocrine cells, potentially linked to hormone secretion and/or metabolic regulation. Autophagic activity is essential for maintaining pancreatic β-cell function, endoplasmic reticulum homeostasis, and immune tolerance. Defective autophagy in mouse β-cells has been shown to cause metabolic stress, alter antigen presentation, and increase susceptibility to immune-mediated destruction (Austin et al. 2025). Thus, the pronounced Rab24 expression in islet cells may reflect its involvement in lysosome-related processes that support protein turnover and protection from stress-induced dysfunction.

In the lung, Rab24 staining was elevated in both the alveolar lining cells and the bronchiolar epithelium of 7-day-old mice. The alveolar lining appeared denser in the young animals, with stronger Rab24 staining compared to older mice. Active alveolar remodelling and expansion during early postnatal development coincide with the elevated Rab24 staining in the 7-day-old tissue (Negretti et al. 2021). In contrast, Rab24 staining in the club cells of the bronchiolar epithelium remained consistently high in both age groups tested. These cells synthesise and secrete the lining fluid of the respiratory epithelium (Blackburn et al. 2023). In 1-month-old mice, bronchiolar epithelium also showed immunoreactivity for the markers of autophagy and endosomal-lysosomal system, indicating that in adult mice, these cells are active in these processes.

In other organs, Rab24 levels were more stable across all age groups. In the kidney, Rab24 was uniformly stained in the proximal convoluted tubular epithelial cells and parietal epithelial cells of the Bowman’s capsule, but not in podocytes in 1-month-old and older tissue. The main function of the proximal epithelial cells is reabsorption of water, salts, plasma proteins and essential nutrients from the filtrate originating from glomeruli (Hall 2025). These cells showed positive IHC staining for LC3, Beclin1, Rab5, Rab7, and LAMP1/2. Thus, these cells possess active autophagic, endocytic and lysosomal systems where Rab24 has reported functions (Amaya et al. 2016; Munafo and Colombo 2002; Yla-Anttila et al. 2015). Greater variability in Rab24 staining was observed in the kidney tubules of 7-day-old mice. Stronger staining was observed particularly in larger, more differentiated tubules, showing that Rab24 protein level increases with epithelial cell differentiation.

The differences in Rab24 levels between 7 and 14-day-old and older animals suggest that Rab24 is developmentally regulated. Because pathways such as autophagy, endocytosis, mitosis, and cell migration, where Rab24 has documented roles (Amaya et al. 2016; Chen et al. 2017b; Militello et al. 2013; Yla-Anttila et al. 2015; Figs. 8–10), are essential for both differentiation and maintenance, Rab24 may contribute to these processes throughout life rather than having distinct functions at different stages. During early postnatal development, Rab24 may contribute to neuronal differentiation and/or maintenance, epithelial remodelling, and organ maturation. In adulthood, the role of Rab24 may shift towards maintenance of homeostasis by regulating similar mechanisms. Future research is needed to reveal the exact tissue-specific functions of Rab24 in these processes.

Rab24 staining in different types of epithelial cells, including ependyma, kidney tubular epithelium, bronchiolar epithelium, and pancreatic islet cells, suggests a possible role in the biology of this cell type. Epithelial cells undergo continuous turnover and require tightly regulated intracellular trafficking processes, including endocytosis and autophagy, to maintain tissue integrity (Schwarz et al. 2007; Wong et al. 2019). In line with this, Rab24 contributes to cell proliferation (Fig. 10) (Militello et al. 2013). Many epithelial cells also have active secretory functions: ependymal cells secrete the cerebrospinal fluid, bronchiolar epithelium secretes the lining fluid, and pancreatic islet cells secrete hormones. Epithelial cells can also be active in endocytosis such as the kidney proximal tubule epithelium. Further studies are needed to define the precise contributions of Rab24 to epithelial cell functions.

In several tissues, Rab24 staining closely resembled that of Rab7. This is in line with the published results showing that Rab24 regulates endocytic trafficking by interacting with Rab7 (Amaya et al. 2016). In addition, both Rab24 and Rab7 have been shown to function at a late stage of the autophagic degradation pathway (Jager et al. 2004; Yla-Anttila et al. 2015).

### Rab24 protein levels in cancers

Tissues that showed transient peaks in Rab24 expression during development, such as liver and pancreas, are the same organs where we observed Rab24 alterations in cancers. This parallel suggests that Rab24 may be part of a developmental program reactivated during tumourigenesis, supporting cell proliferation and migration similarly to its proposed functions in organ maturation.

The dynamic changes in Rab24 levels observed during postnatal development in mice, particularly surges coinciding with rapid growth and organ maturation, may mirror mechanisms exploited or disrupted during tumourigenesis. Processes regulated by Rab24, such as endocytosis, autophagy, as well as mitosis, cell migration, and invasion could be exploited by cancer cells to support their growth, survival, and metastasis (Amaya et al. 2016; Chen et al. 2017b; Militello et al. 2013; Yla-Anttila et al. 2015; Figs. 9, 10). In line with this, RNA sequencing of Rab24-deficient Neuro-2a cells revealed reduced expression of genes associated with extracellular matrix organization, angiogenesis, cell differentiation, regulation of transcription, and signal transduction, further linking Rab24 to pathways that govern both developmental growth and tumour progression. Furthermore, the tissue-specific dynamic variations in Rab24 levels with age emphasize the importance of context in the possible roles of Rab24. Dysregulation in cancer may arise from perturbations in the finely-tuned developmental programs (Stanger and Wahl 2024).

In hepatocytes, RAB24 overexpression promotes cell motility, invasion, and adhesion, accelerates cell cycle progression, epithelial to mesenchymal transition, and reduces apoptosis, while knockdown of RAB24 has opposite effects (Chen et al. 2017b). In mouse liver, Rab24 levels were high 14 days after birth but then decreased and stayed low (Fig. 2e). The liver weight of mice starts to rapidly increase at the same age of two weeks (Moreno-Carranza et al. 2018). In adult liver, RAB24 level is low in humans (Chen et al. 2017b, Fig. S7g) and mice (Figs. 2e, 6f), but RAB24 is elevated in HCC and enhances the malignant phenotype (Chen et al. 2017b). Thus, high Rab24 levels correlate with increased cell proliferation and migration both during mouse development and in human cancer. Consistent with these observations, we show here that similar to hepatocyte cell lines (Chen et al. 2017b), Rab24 knockout reduced proliferation and migration in Neuro-2a and HeLa cells (Figs. 9, 10), indicating that Rab24 supports cellular proliferation and motility across different cell types.

Because the mouse brain exhibited the highest Rab24 protein levels among the tissues, we analysed whether RAB24 is also altered in human tumours of neuronal origin. Analysis of a brain cancer TMA revealed that RAB24 protein levels varied across tumour types. Medulloblastomas showed the highest RAB24 H-scores, significantly exceeding those of normal adjacent brain tissue, whereas astrocytomas, glioblastomas, oligodendrogliomas, ependymomas, and meningiomas, cancers originating from non-neuronal cell types, displayed staining intensities comparable to normal brain tissue. In neuroblastomas, tumours of peripheral neuronal origin, RAB24 staining was also significantly stronger than in normal peripheral nerve tissue. Among the subtypes, INSS stage I and poorly differentiated neuroblastomas exhibited the highest RAB24 levels. Moreover, compared to normal peripheral nerve, RAB24 levels were higher in neuroblastomas of children. These findings suggest that RAB24 protein expression is upregulated in specific neuronal cancers, possibly reflecting reactivation of developmental expression programs that are normally high in neurons during maturation.

The preferential upregulation of RAB24 in medulloblastomas and neuroblastomas, but not in glial-derived brain tumours, suggests that RAB24 protein expression could reflect neuronal lineage or differentiation state rather than a general oncogenic feature. Future studies combining RAB24 expression profiling with neuronal differentiation markers could clarify whether high RAB24 defines a subset of tumours maintaining neuronal identity. However, rather than reflecting developmental immaturity alone, the RAB24 expression pattern may also indicate increased autophagic and vesicle trafficking demands in rapidly proliferating or metabolically active tumour cells. Such features are typical of paediatric tumours and poorly differentiated cancers, which often rely on efficient autophagy to sustain growth and survival under stress (Gatto et al. 2021; Zhang et al. 2025). In medulloblastoma in particular, inhibition of autophagy has been shown to suppress tumour growth, reduce tumorigenicity and impair metastatic potential, underscoring the importance of autophagic flux in maintaining tumour cell survival and progression (Bharambe et al. 2019; Singh et al. 2017).

We observed a peak in Rab24 levels in the pancreas at 7 and 14 days of age (Fig. 2d). IHC showed highest Rab24 signals in the pancreatic islets, with some signals in the exocrine tissue at 7 days (Fig. 5g, h). In mice, pancreatic islets grow and mature after birth. The β-cell specific urocortin 3 is upregulated starting at postnatal day 6 (Blum et al. 2012). Thus, start of β-cell differentiation coincides with the peak in Rab24 levels (Fig. 2d). The islets showed Rab24 staining in IHC in both age groups tested (Fig. 5g, h). Moreover, in PNET samples, which originate from islets, Rab24 staining was reduced compared to normal islets, and Rab24 staining was lower in more advanced cancer stages (G2 and G3) than in early stage (G1) as assessed by the Ki-67 index (Fig. 13e). Although the number of G2 and G3 samples in our cohort was limited, our findings suggest that RAB24 protein downregulation may be linked to more aggressive tumour phenotypes. In line with this, Yau et al. showed that RAB24 is among the proteins downregulated when β-cells lose their identity due to loss of vacuolar protein sorting 41 (VPS41), a protein required in insulin secretion in β-cells (Yau et al. 2025, Supplemental Table 1). These findings suggest that Rab24 protein level reflects islet identity in both developing mouse pancreas and in human PNET. Future studies with larger patient cohorts should explore whether RAB24 levels correlate with clinical parameters such as patient survival or response to therapy, and whether loss of RAB24 contributes functionally to increased malignancy.

The mammalian target of rapamycin complex 1 (mTORC1) nutrient sensing kinase complex is a central regulator of cell growth and metabolism and mediates postnatal islet maturation in mice (Sinagoga et al. 2017). mTORC1 activation is common in PNETs (Werle et al. 2023), and mTORC1 inhibitors are in clinical trials for PNET treatment (Rajdev et al. 2022). Thus, high mTORC1 activity is common for islet development and PNET. Both mTORC1 and Rab24 are implicated in autophagy: mTORC1 activity inhibits autophagy (Dossou and Basu 2019), and Rab24 is required for clearance of late autophagic compartments (Yla-Anttila et al. 2015). Although no direct molecular links between mTORC1 and Rab24 have yet been established, their complementary roles in growth regulation and autophagy suggest potential functional interplay. Further research should investigate whether Rab24 modulates autophagic or lysosomal functions downstream of mTORC1 signalling and whether its loss contributes to PNET pathogenesis.

### Cancer-specific differences in RAB24 protein expression

Our results showed that RAB24 levels varied markedly among human cancers, being elevated in breast and skin cancers, neuroblastomas, and medulloblastomas, but reduced in tumours of the digestive system and the urinary tract, and PNET. The observed heterogeneity in RAB24 levels may reflect the different cell types from which the cancers originate, and the multifaceted cellular functions of Rab24, which may be hijacked or altered in cancer cells to support oncogenic processes. The analysis of *RAB24* mRNA levels and patient survival in the TCGA datasets further revealed context-dependent expression of RAB24 in cancer. In HCC cell lines, elevated RAB24 levels promote proliferation, migration, and epithelial-mesenchymal transition, enhancing invasive and metastatic capacity by promoting malignant phenotypes such as vasculogenic mimicry and cell adhesion (Chen et al. 2017b). Patients with high *RAB24* mRNA expression in tumours also had reduced overall survival (Fig. S16b), in agreement with published results on this cancer type (Chen et al. 2017b; Gu et al. 2025; He et al. 2002; Yang et al. 2020; Zhang et al. 2020; Zhu et al. 2020). High RAB24 is also an unfavourable prognostic marker in prostate cancer (Hu et al. 2020). Conversely, in PAAD, RAB24 is an independent low-risk factor, its reduced expression correlates with improved patient outcome (Deng et al. 2022, Yu et al. 2021). *RAB24* mRNA levels were significantly lower in tumour tissues than in normal pancreas (Fig. S16a), consistent with our multicancer TMA IHC results (Table S2). Survival analysis revealed that high *RAB24* expression was associated with significantly improved overall survival in PAAD patients (Fig. S16a) in agreement with published results. In lung squamous cell carcinoma, RAB24 IHC staining did not differ between tumour and normal tissue and mRNA levels showed no association with patient outcome. These opposing associations show that RAB24’s correlation with patient outcome depends on the cancer-specific context.

Such dual cancer-type dependent behaviour is not unique to RAB24. Several RAB GTPases show altered expression levels and context-dependent roles in cancer (Ji et al. 2025). For instance, RAB25 can act as an oncogene in ovarian and prostate cancers but suppress invasion in colon carcinoma (Cho et al. 2024, Hu et al. 2017, Wang et al. 2017). Similarly, RAB27A promotes metastatic behaviour in some cancers but may limit the invasion in others (Kren et al. 2020).

At the molecular level, RAB24 may influence tumour behaviour through its established functions in cell proliferation and migration, and in the endosomal and autophagic pathways. Autophagy has a well-known dual role in cancer; supporting early tumour suppression but later promoting cell survival under stress (Rakesh et al. 2022, Yun et al. 2021). Rab24 facilitates the clearance of late autophagic compartments (Yla-Anttila et al. 2015), and its dysregulation could therefore shift the balance between beneficial and detrimental autophagy depending on tissue context. Moreover, RAB proteins regulate aspects of the tumour microenvironment, including antigen presentation, extracellular matrix organization, and cell–cell communication (Ji et al. 2025). In line with this, our RNA sequencing suggested possible roles for Rab24 in signal transduction, gene expression, immunity, and extracellular matrix organization, all of which can influence cancer progression.

RAB24 may participate in growth and differentiation programs that can be reactivated or suppressed during tumourigenesis in a tissue-specific manner. The same developmental pathways that regulate organ maturation in early life may be exploited by cancer cells to promote proliferation, invasion or metabolic adaptation. Further studies combining functional experiments with transcriptomic and proteomic analyses across cancer types are needed to elucidate how RAB24 contributes to these context-dependent outcomes and to assess its potential as a diagnostic or therapeutic target in cancer.

### Conclusions and implications

In this study, we systematically characterized age-, tissue-, and cell-type-specific Rab24 protein levels in mouse tissues by western blotting and IHC. The dynamic changes in Rab24 protein levels during early development suggest possible roles in organ maturation, particularly in the brain, heart, skeletal muscle, pancreas and liver whereas its stable levels in adulthood may reflect possible maintenance functions in differentiated cells such as neurons, kidney tubule epithelium and pancreatic islet cells.

In the cancer context, RAB24 protein levels displayed distinct, cancer-specific alterations. RAB24 was increased in breast and skin cancers compared to corresponding normal tissues, while the opposite was found in cancers of the digestive system and the urinary tract. In the brain cancer and neuroblastoma TMAs, RAB24 levels were elevated in medulloblastoma and neuroblastoma that both originate from neuronal cells. In pancreatic PNET, RAB24 levels were lower than in normal islet cells, and reduced RAB24 levels were found in higher grade tumours, suggesting that RAB24 loss may be associated with more aggressive tumour behaviour. These observations suggest that RAB24 protein level is regulated in a context-dependent manner that may mirror its developmental roles.

Although Rab24 protein levels do not directly indicate functional significance, our findings highlight Rab24 as a potential regulator of cell proliferation, migration, differentiation, and homeostasis, processes that are relevant to both development and tumourigenesis. Future studies should focus on defining the molecular mechanisms and interaction partners of Rab24, its tissue-specific functions, and its potential as a diagnostic and therapeutic target in human disease.

## Supplementary Information

Below is the link to the electronic supplementary material.

ESM 1 (PDF 5.80 MB)

ESM 2 (XLSX 16.7 KB)

ESM 3 (XLSX 19.3 KB)

ESM 4 (XLSX 15.9 KB)

ESM 5 (XLSX 13.5 KB)

## Data Availability

No datasets were generated or analysed during the current study.
