# Supplementary material for: Rab24 protein levels show dynamic changes in mouse tissues and human cancers
Source: Cell Tissue Res. 2026 Jan 27;403(2):14. doi: 10.1007/s00441-025-04043-4 (PMC12847187; doi:10.1007/s00441-025-04043-4)
Supplement: Supplementary file 1 — (PDF 5.80 MB) [file 441_2025_4043_MOESM1_ESM.pdf]

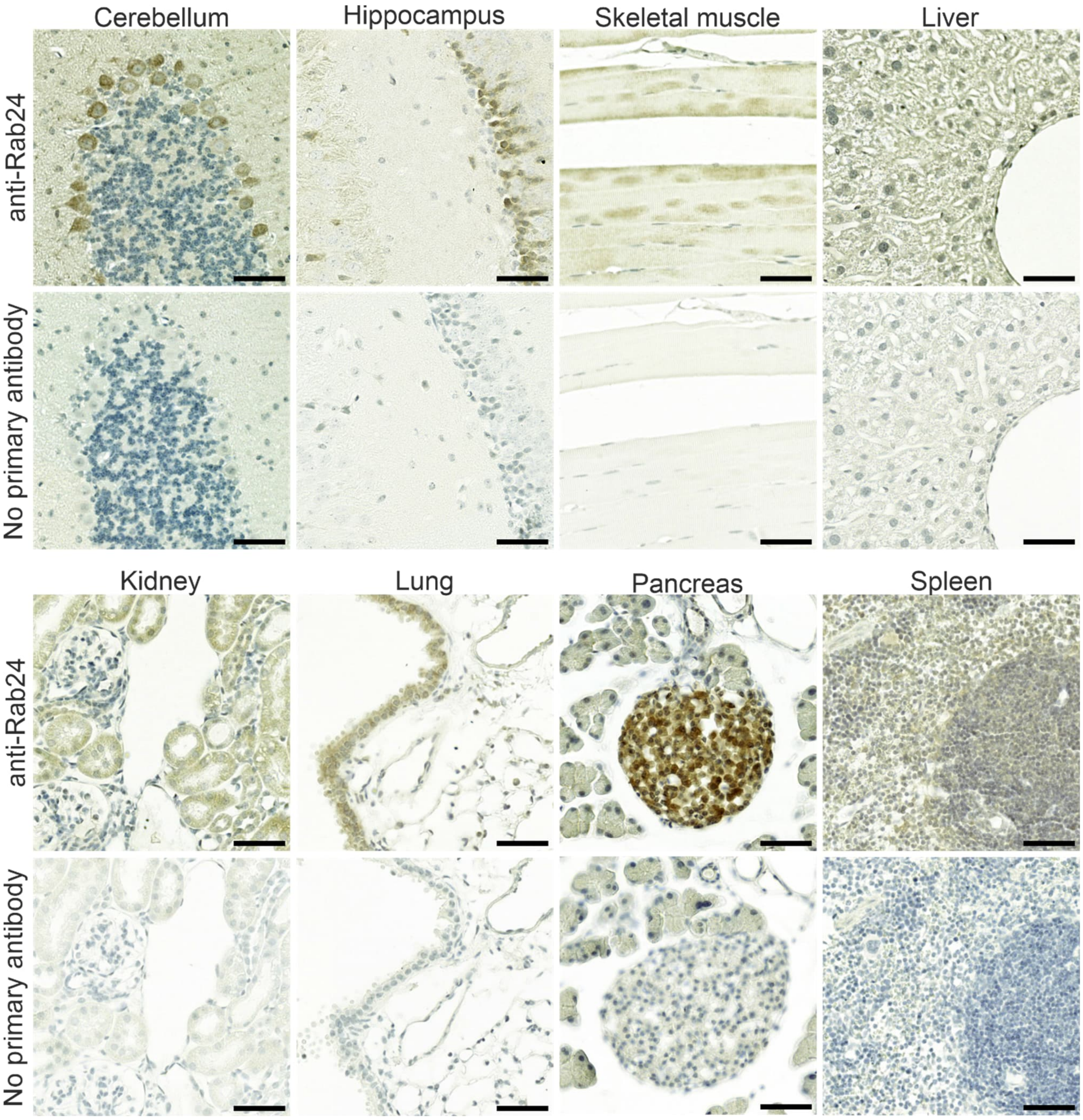

Figure S1.

Fig. S1 Immunohistochemical staining with and without Rab24 antibody in various mouse tissues. The two staining protocols were performed using consecutive serial sections. Representative images are shown for anti-Rab24 and the corresponding control sections, as indicated on the left. Positive Rab24 staining is visible as brown colour in the anti-Rab24-stained sections. Scale bars: 50 µm.

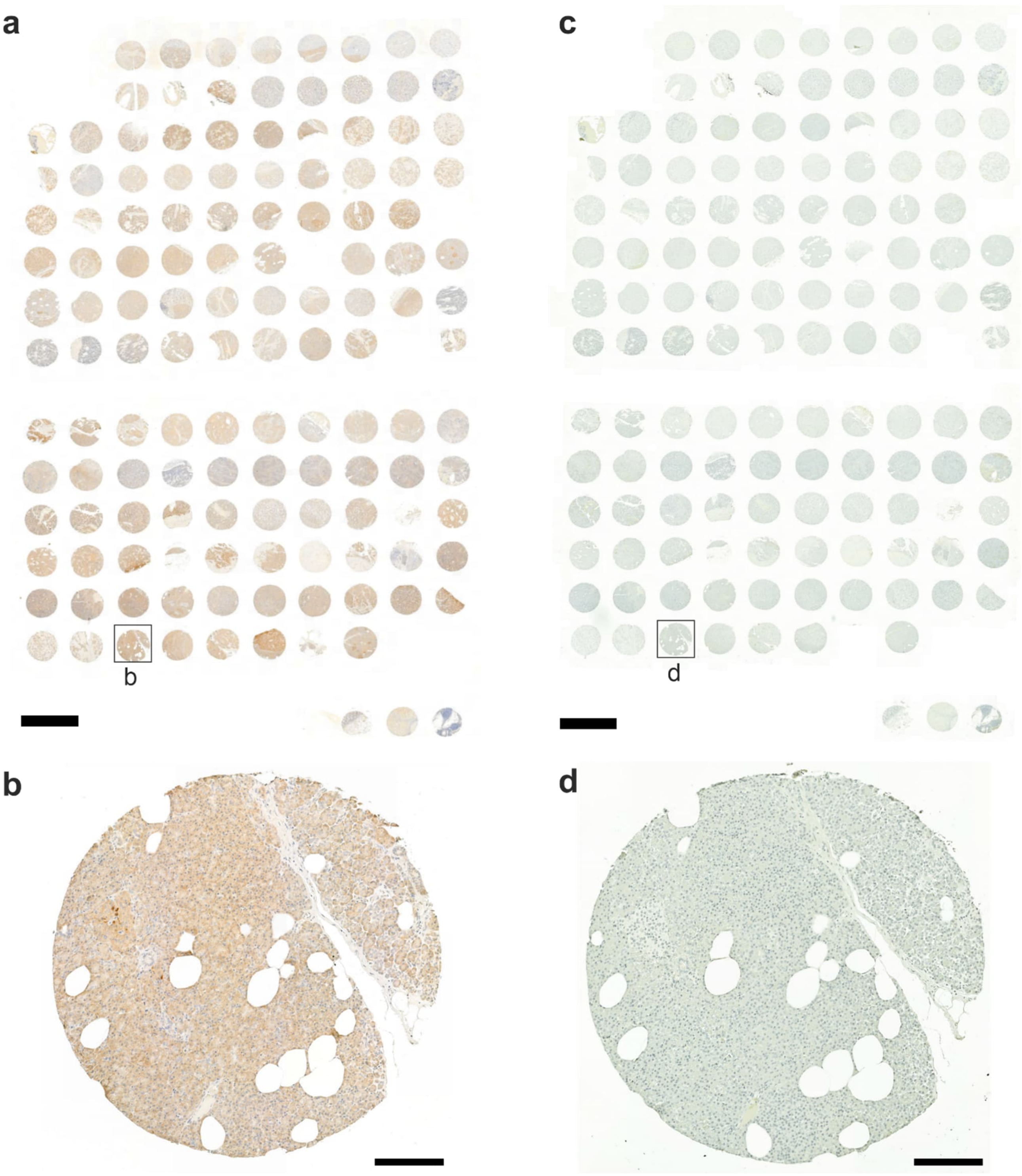

Figure S2.

Fig. S2 Immunohistochemical staining of RAB24 in the tissue microarray containing samples of human pancreatic neuroendocrine tumours and pancreatic normal tissue. Representative images are shown for anti-RAB24 staining (**a, b**) and the serial control section stained without the primary antibody (**c, d**). RAB24 staining is visible as brown colour in the anti-RAB24-stained tissue cores. Scale bars: 2 mm (**a, c**) and 200 µm (**b, d**).

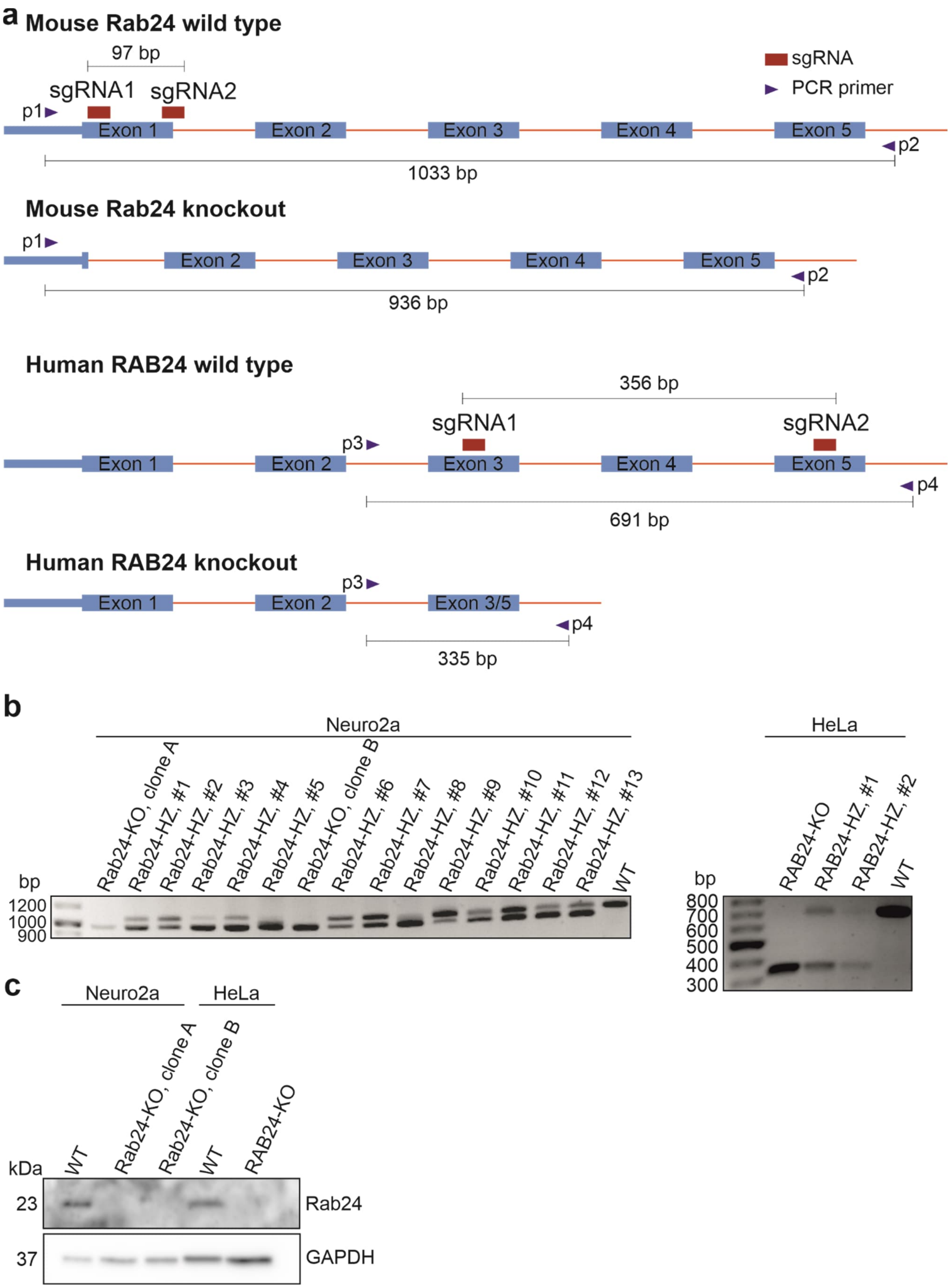

Figure S3.

Fig. S3 Generation and validation of Rab24-knockout (KO) cell lines. (**a**) Schematic representation of the CRISPR/Cas9-mediated knockout strategy used to generate Neuro-2a and HeLa Rab24-KO

cell lines. Guide RNA target sites and PCR primer binding sites within the *RAB24* coding sequence are indicated. (**b**) Screening of potential KO clones was performed using PCR genotyping, followed by agarose gel electrophoresis of the PCR products amplified using the primers listed in Table S7. Representative gels show the expected PCR fragments for wild type (WT), heterozygous (HZ), and knockout (KO) clones in Neuro-2a (left) and Hela (right) cells. (**c**) Western blot validation of Rab24 protein depletion in cell extracts from WT and KO clones of Neuro-2a and HeLa cells.

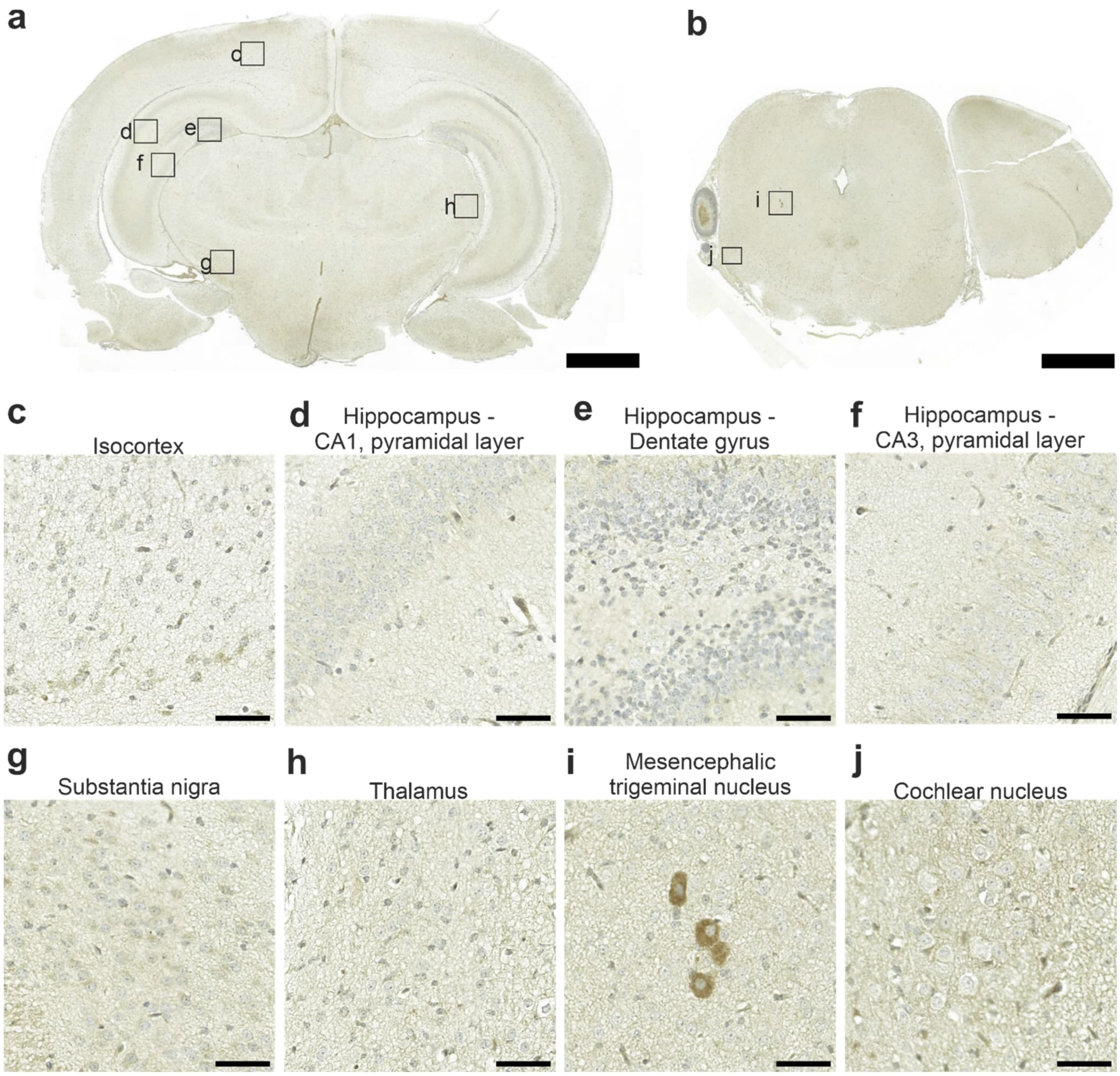

Figure S4.

Fig. S4 Rab24 immunohistochemical staining in the isocortex, hippocampus, midbrain and hindbrain of 7-day-old mice. The boxed areas in panels **a** and **b** indicate the regions shown at higher magnification in panels **c-j**. The brown colour (panel **i**) indicates Rab24-positive staining. Sections from four mice (three males and one female) were used for the analysis. Representative images are shown. Scale bar: 1 mm (**a, b**) and 50 µm (**c-j**).

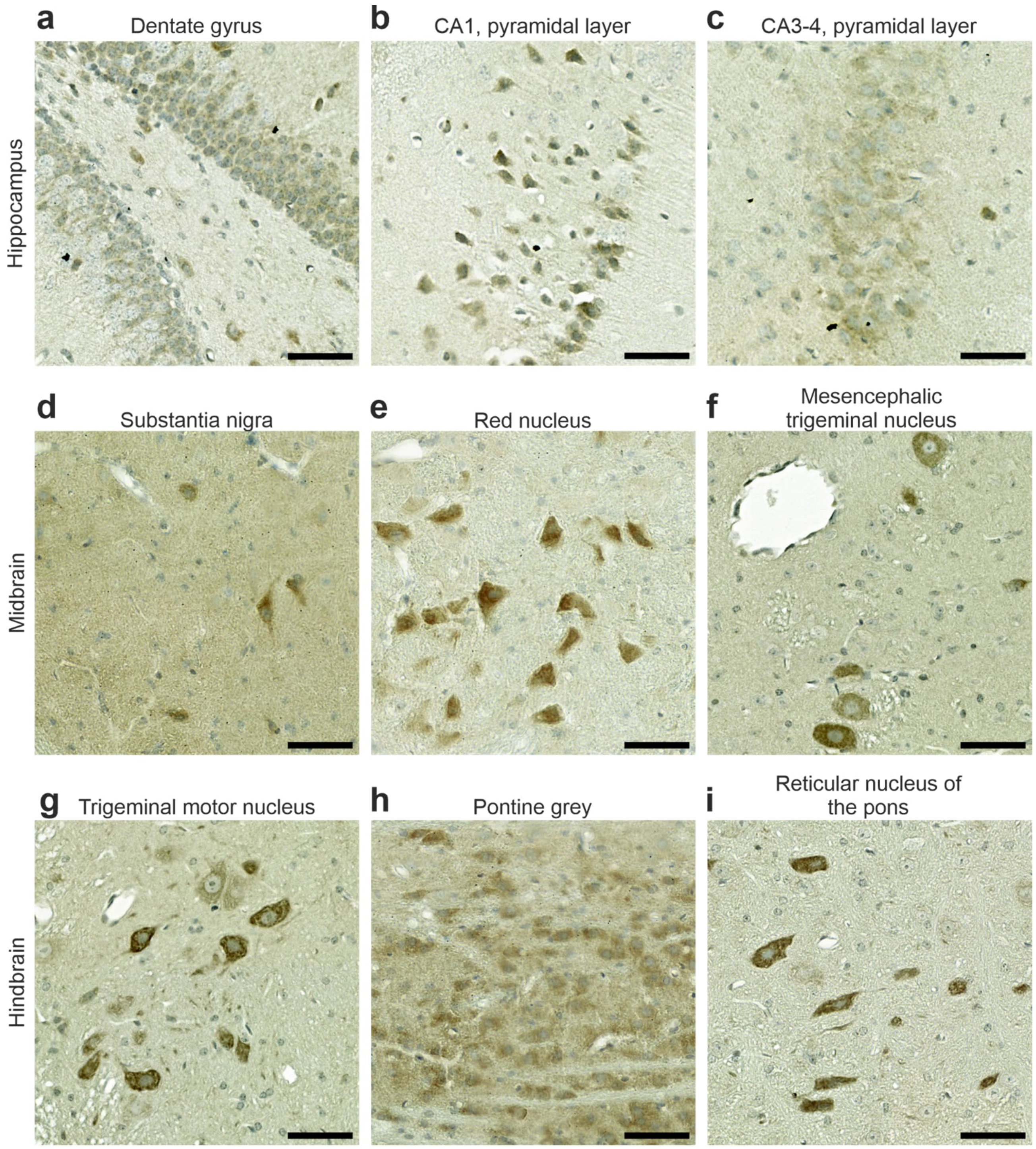

Figure S5.

Fig. S5 Rab24 immunohistochemical staining in the brain of 3-month-old mice (**a-i**). Images show the hippocampus, midbrain and hindbrain regions. Brown Rab24-positive cells correspond to neurons. Staining was performed on sections from eight animals (four males and four females); representative images are shown. Scale bar: 50 µm.

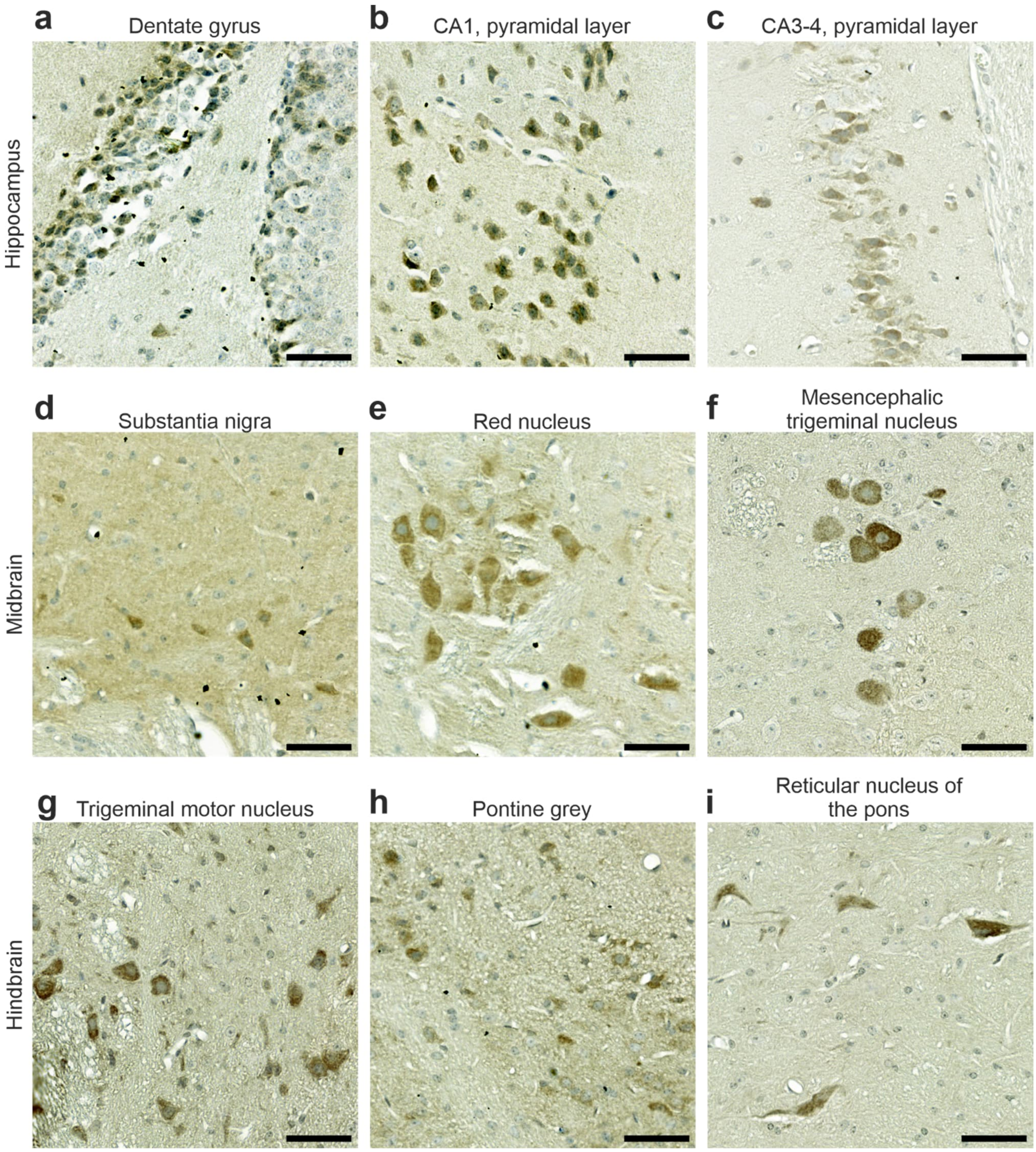

Figure S6.

Fig. S6 Rab24 immunohistochemical staining in the brain of 6-month-old mice (**a-i**). Images show the hippocampus, midbrain and hindbrain regions. Brown Rab24-positive cells correspond to neurons. Staining was performed on sections from eight animals (four males and four females); representative images are shown. Scale bar: 50 µm.

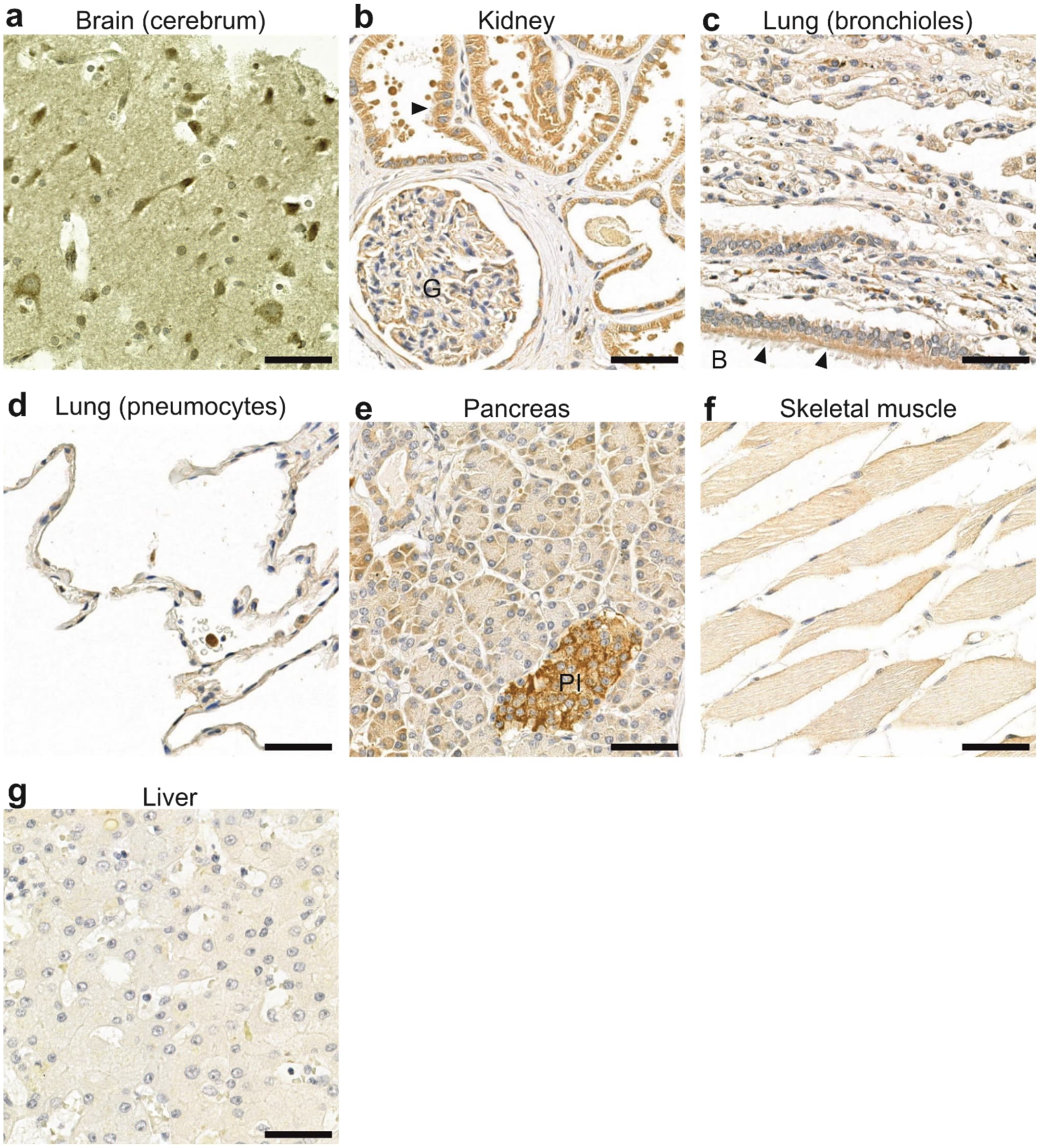

Figure S7.

Fig. S7 RAB24 immunohistochemical staining in adult human tissues. (**a**) Neurons showing brown RAB24-positive staining. (**b**) Arrowhead indicates RAB24-positive epithelial cell lining the kidney tubular cells. (**c**) Arrowheads indicate RAB24-positive cells in the bronchiolar epithelium. (**d-g**) Lung, pancreas, skeletal muscle and liver are also shown. G, kidney glomerulus; B, bronchiole lumen; PI, pancreatic islet. Scale bar: 50 µm.

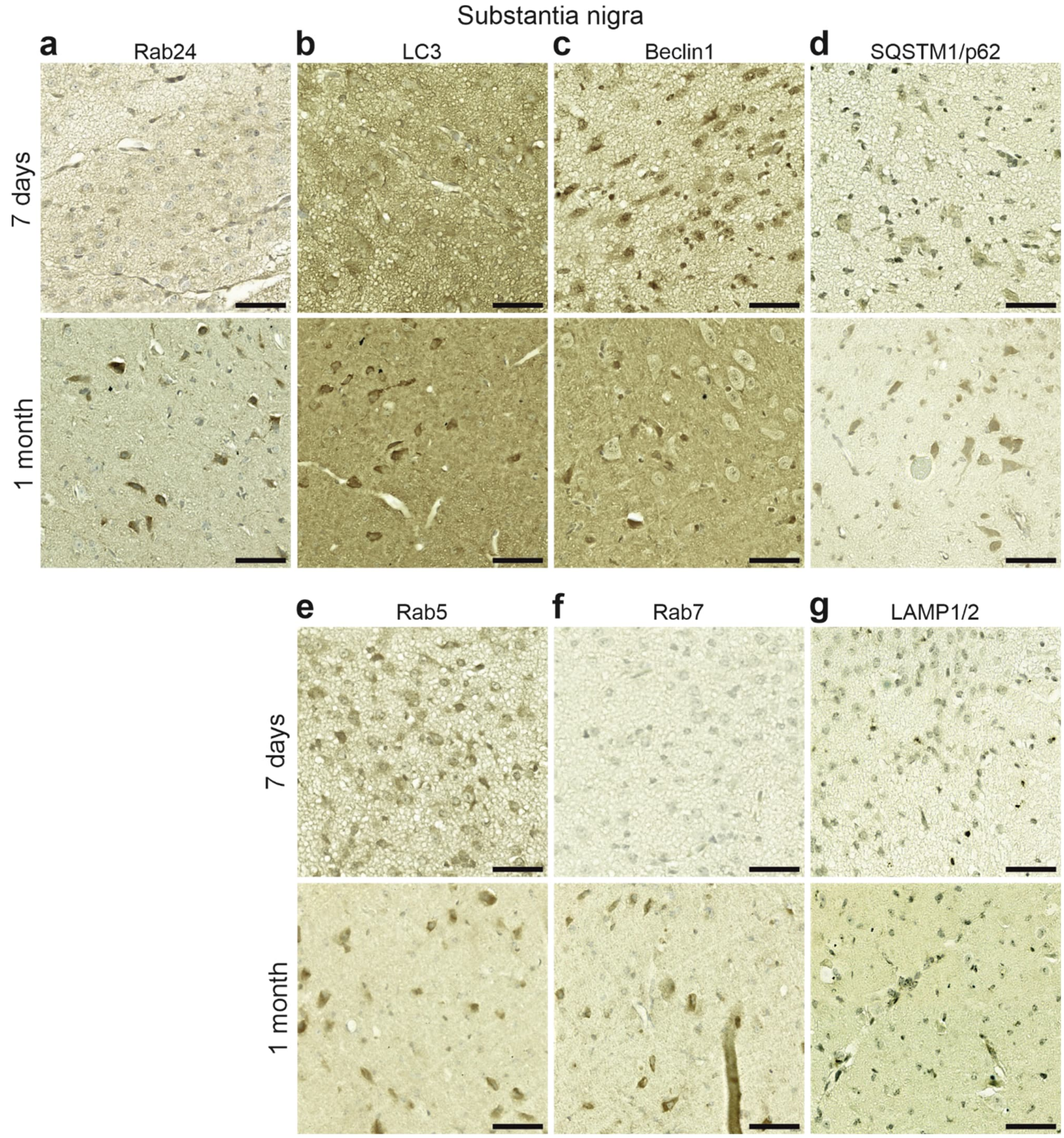

Figure S8.

Fig. S8 Staining of Rab24, autophagy markers LC3, Beclin1, and SQSTM1/p62, endosomal markers Rab5 and Rab7, and lysosomal markers LAMP1 and LAMP2 (LAMP1/2) in mouse substantia nigra (**a-g**). The staining was performed on sections from two animals for 7-day-old mice and two animals for 1-month-old mice; representative images are shown. Scale bar: 50 µm.

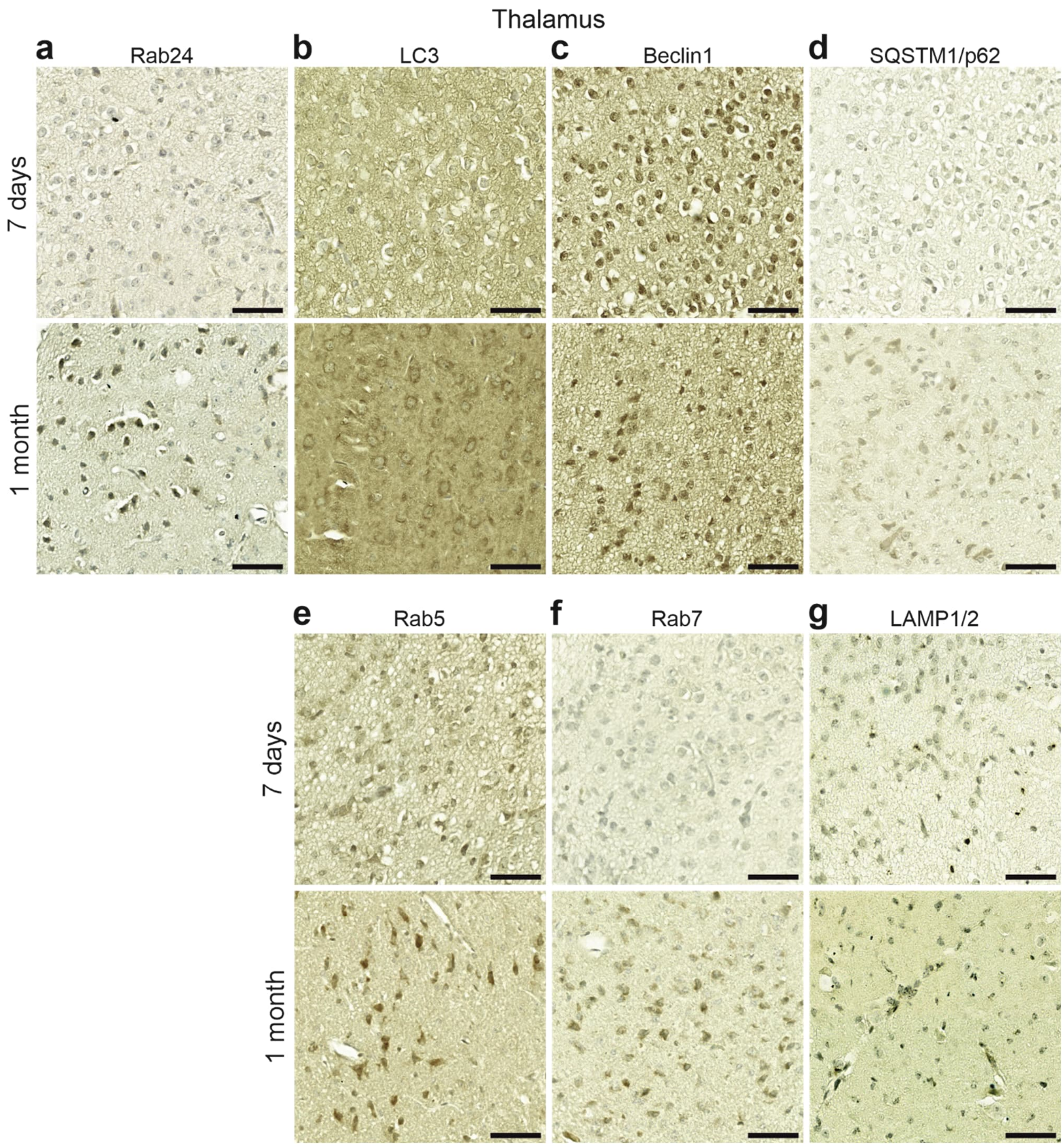

Figure S9.

Fig. S9 Staining of Rab24, autophagy markers LC3, Beclin1, and SQSTM1/p62, endosomal markers Rab5 and Rab7, and lysosomal markers LAMP1 and LAMP2 (LAMP1/2) in mouse thalamus (**a**-**g**). The staining was performed on sections from two animals for 7-day-old mice and two animals for 1-month-old mice; representative images are shown. Scale bar: 50 µm.

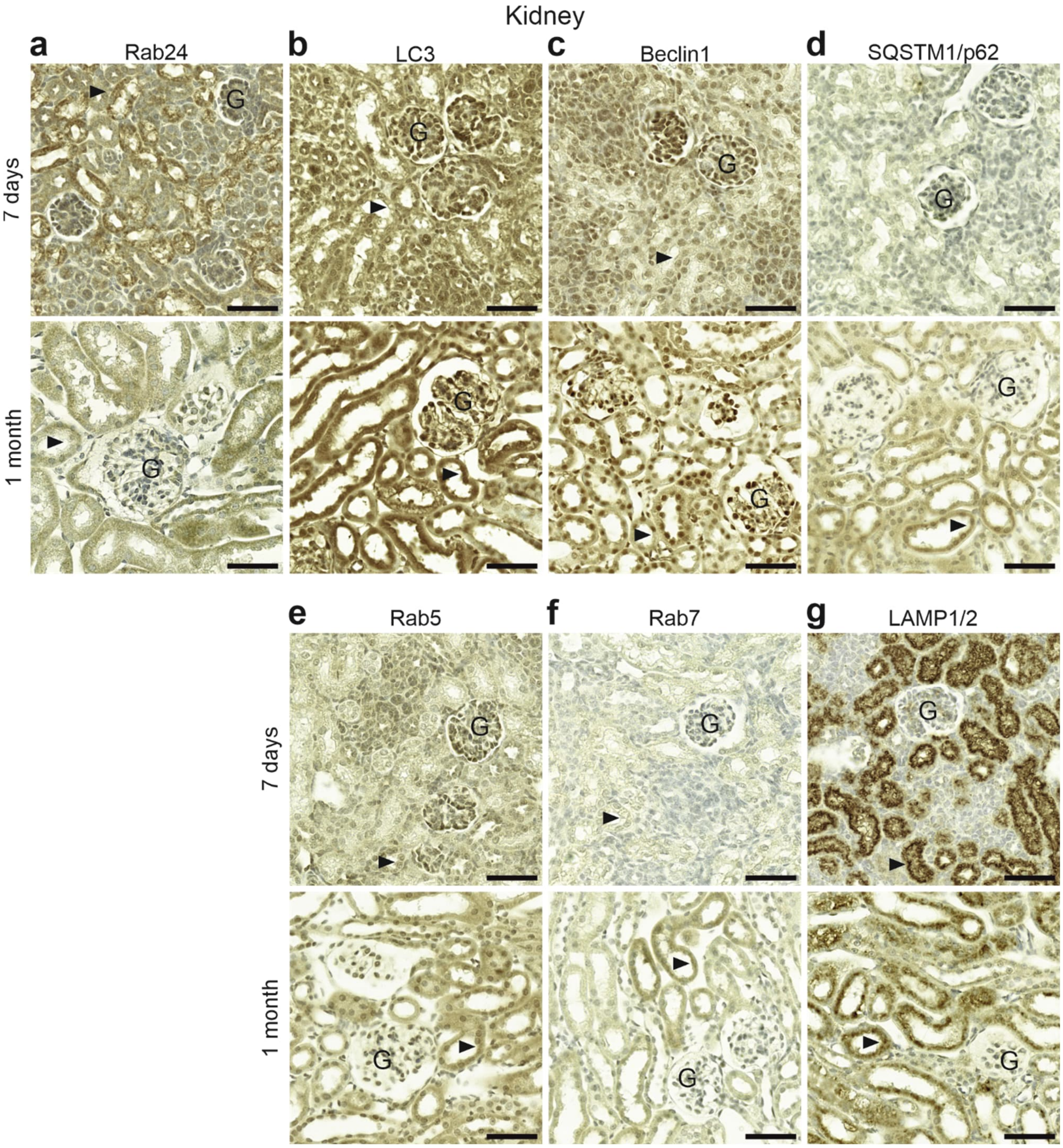

Figure S10.

Fig. S10 Staining of Rab24, autophagy markers LC3, Beclin1, and SQSTM1/p62, endosomal markers Rab5 and Rab7, and lysosomal markers LAMP1 and LAMP2 (LAMP1/2) in mouse kidney (**a-g**). Representative images of 7-day-old and 1-month-old mice are shown. Arrowheads indicate epithelial cells lining the kidney tubules. G, kidney glomerulus. The staining was performed on sections from two animals for 7-day-old mice and two animals for 1-month-old mice. Scale bar: 50 μm.

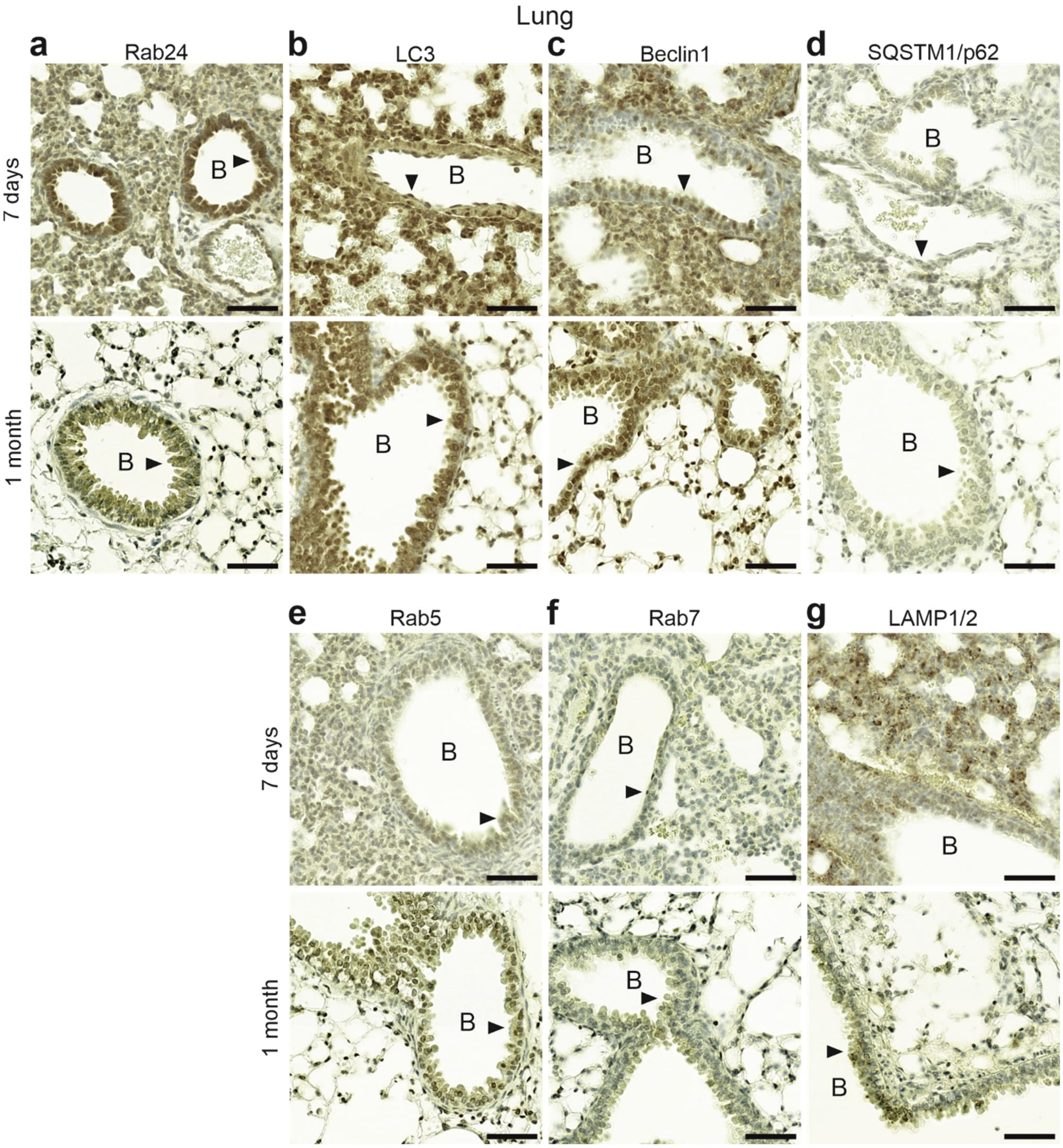

Figure S11.

Fig. S11 Staining of Rab24, autophagy markers LC3, Beclin1, and SQSTM1/p62, endosomal markers Rab5 and Rab7, and lysosomal markers LAMP1 and LAMP2 (LAMP1/2) in mouse lung (**a-g**). Representative images of 7-day-old and 1-month-old mice are shown. Arrowheads indicate epithelial cells lining the bronchiolar epithelium. B, bronchiole lumen. The staining was performed on sections from two animals for 7-day-old mice and two animals for 1-month-old mice. Scale bar: 50 μm.

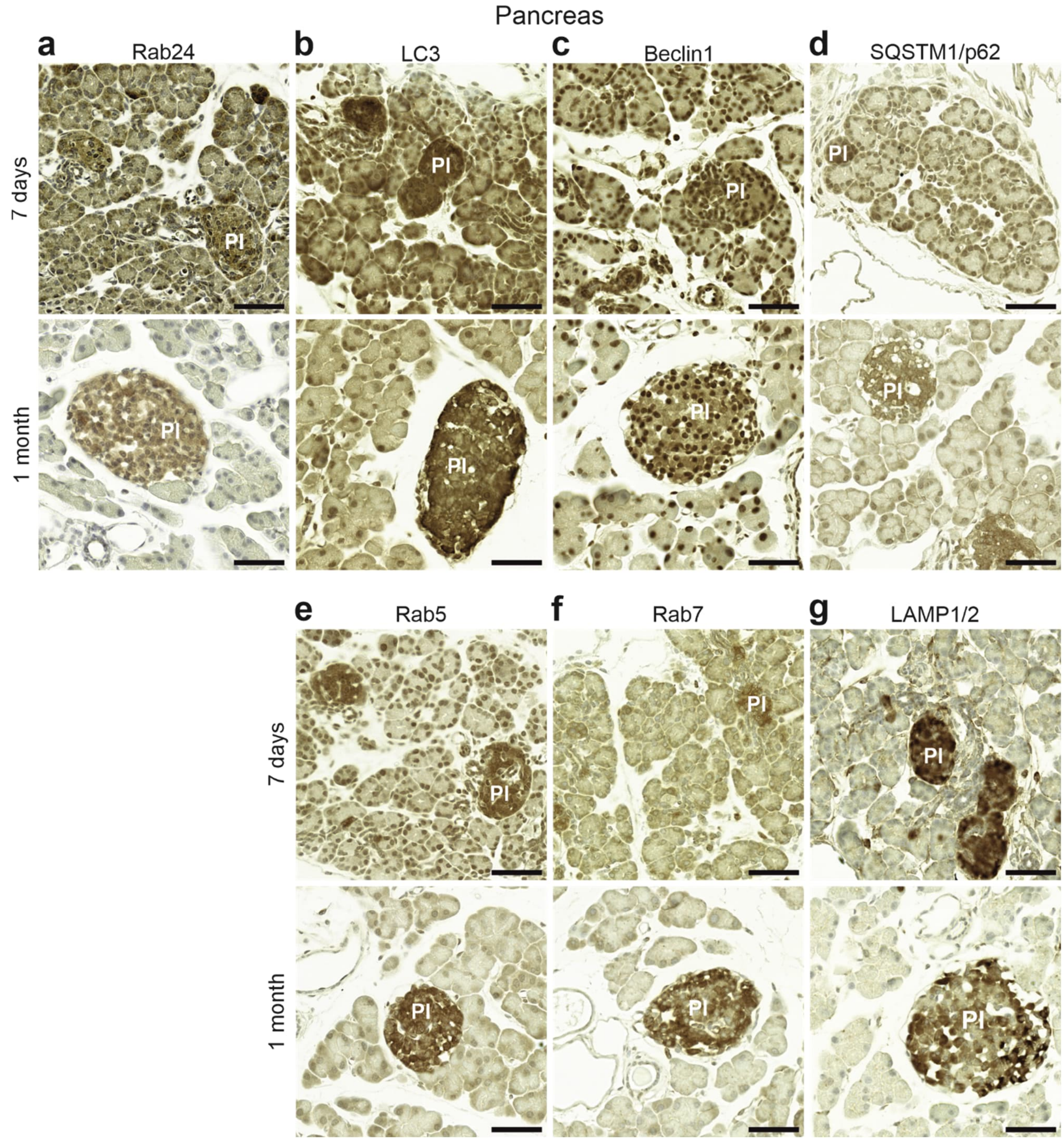

Figure S12.

Fig. S12 Staining of Rab24, autophagy markers LC3, Beclin1, and SQSTM1/p62, endosomal markers Rab5 and Rab7, and lysosomal markers LAMP1 and LAMP2 (LAMP1/2) in mouse pancreas (**a-g**). Representative images of 7-day-old and 1-month-old mice are shown. PI, pancreatic islet. The staining was performed on sections from two animals for 7-day-old mice and two animals for 1-month-old mice. Scale bar: 50 μm.

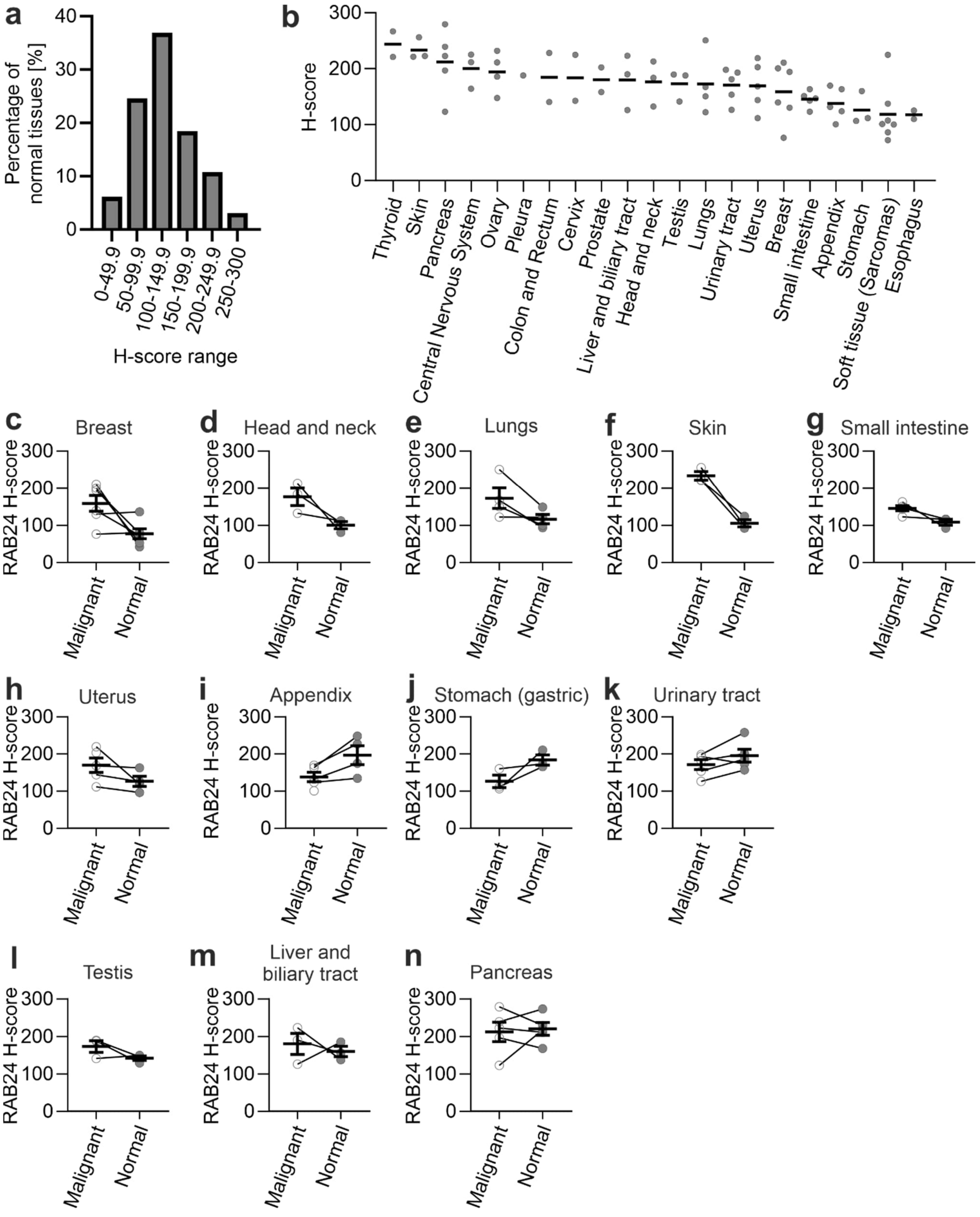

Figure S13.

Fig. S13 Comparison of the H-scores for RAB24 immunohistochemical staining intensities in malignant and normal human tissues grouped by organ or tissue of origin. The analysis was done from the multicancer tissue microarray (Table S2). (**a**) Frequency distribution of RAB24 H-scores in normal tissues removed together with tumour samples during surgery. (**b**) Overview of RAB24 H-scores across 21 categories of cancers grouped by tissue or organ of origin. Individual samples are indicated by the grey dots and the horizontal line indicates the average. (**c–n**) Tissue categories

demonstrating a trend of increased (**c-h**), decreased (**i-k**), or of no change (**l-n**) in RAB24 H-score in malignant versus normal tissues. The horizontal lines indicate mean ± SEM. Statistical significance was determined by Wilcoxon test; none of the differences between malignant and normal tissue in panels **c-n** is statistically significant.

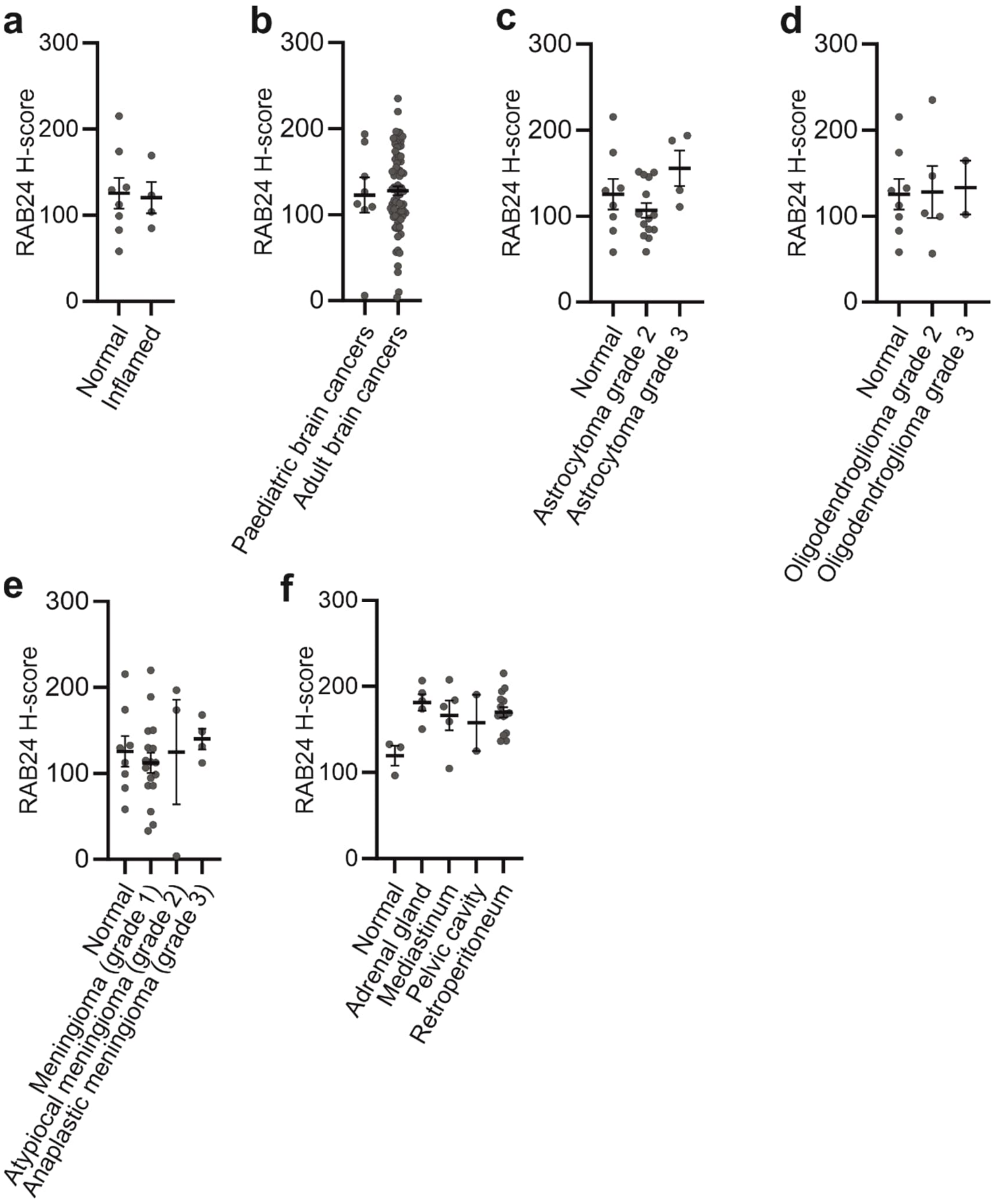

Figure S14.

Fig. S14 Analyses of RAB24 H-scores from the tissue microarrays containing brain tissue and brain cancers (Table S4) and peripheral nerve tissue and neuroblastomas (Table S5). (**a**) Quantification of RAB24 H-scores in normal versus inflamed brain tissue. (**b**) Quantification of RAB24 H-scores in paediatric (4 - 14 years of age) versus adult (17-75 years of age) brain tumours. (**c**) Quantification of RAB24 H-scores in normal brain tissue and astrocytomas of grades 2 and 3. (**d**) Quantification of RAB24 H-scores in normal brain tissue and oligodendrogliomas of grades 2 and 3. (**e**) Quantification of RAB24 H-scores in normal brain tissue and meningiomas of grades 1, 2, and 3. (**f**) Quantification of RAB24 H-scores in neuroblastomas of different anatomical origins (adrenal gland, mediastinum, pelvic cavity, retroperitoneum) compared to normal peripheral nerve. Statistical significance was determined by Mann-Whitney test (**a, b**) and Kruskal–Wallis test with Dunn's post hoc correction (**c, d, e, f**). No significant differences were observed in any of these comparisons.

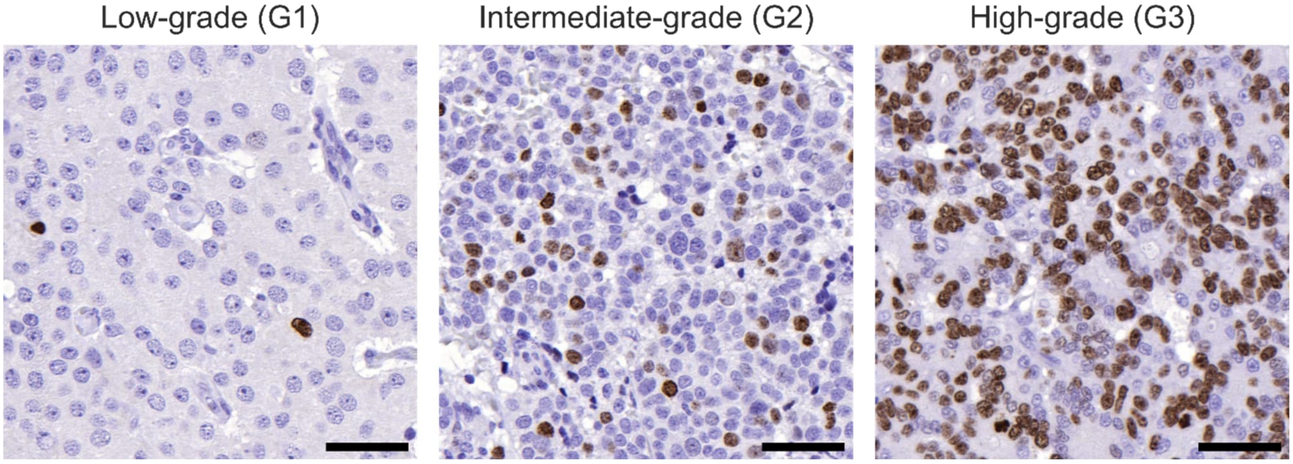

Figure S15.

Fig. S15 Representative immunohistochemistry images of Ki-67 staining in pancreatic neuroendocrine tumour (PNET) samples, illustrating tumour grade classification based on Ki-67 index. G1, 0-3% Ki-67-positive cells; G2, 3-20% Ki-67-positive cells; and G3, more than 20% Ki-67-positive cells.

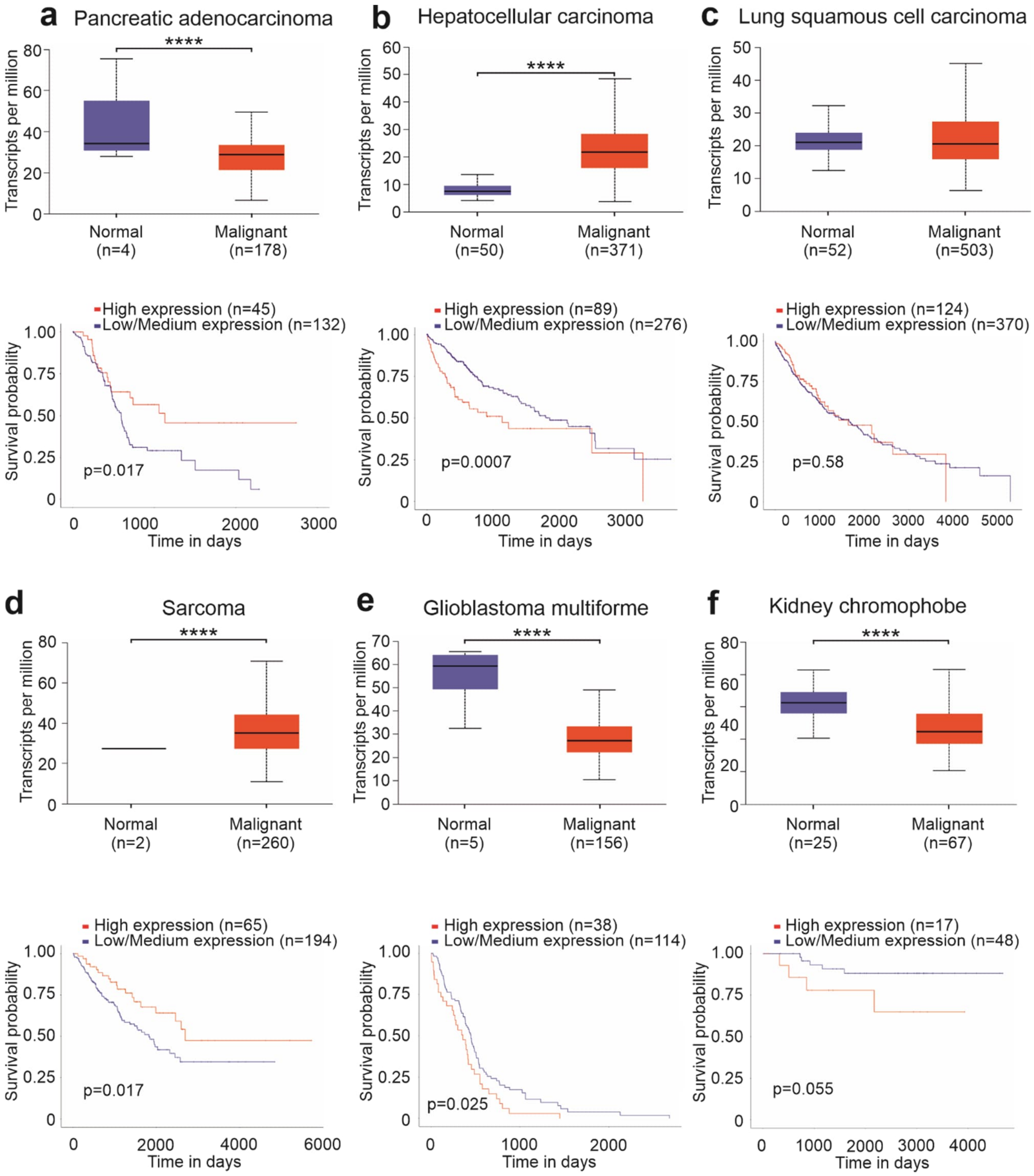

Figure S16.

Fig. S16 *RAB24* mRNA expression levels and patient survival in six cancer types based on The Cancer Genome Atlas (TCGA) data. (**a–f**) *RAB24* mRNA expression levels in normal and tumour tissues (upper panels) and corresponding Kaplan–Meier overall survival analyses (lower panels) for (**a**) pancreatic adenocarcinoma, (**b**) hepatocellular carcinoma, (**c**) lung squamous cell carcinoma, (**d**) sarcoma, (**e**) glioblastoma multiforme, and (**f**) kidney chromophobe carcinoma. mRNA expression data were obtained from TCGA via the UALCAN portal. Box plots represent the 25th and 75th percentiles (boxes), median (line), and minimum and maximum (whiskers) as defined by the UALCAN output. Survival curves compare patients with high versus low/medium *RAB24* mRNA expression, and p-values indicate the results of the log-rank test.

Table S1 List of the primary and secondary antibodies used in this study.

Antigen	Assay	Dilution	Host	Vendor	Product number
Rab24	WB	1:1000	Rabbit	Proteintech	11445-1-AP
GAPDH	WB	1:15000	Mouse	Abcam	ab8245
Mouse IgG, HRP-conjugated	WB	1:20000	Goat	Jackson Laboratory	115-035-003
Rabbit IgG, HRP conjugated	WB	1:15000	Goat	Jackson Laboratory	111-035-003
Rab24	IHC	1:400	Rabbit	Proteintech	11445-1-AP
Rab5	IHC	1:200	Rabbit	Santa Cruz	sc-28570
Rab7	IHC	1:200	Rabbit	Cell Signalling	9367S
p62/SQSTM1	IHC	1:200	Rabbit	Abgent	AP2183b
LC3	IHC	1:200	Rabbit	Novus	NB-100
Beclin-1	IHC	1:200	Rabbit	Novus	NB-500
LAMP1	IHC	1:100	Rat	Developmental Studies Hybridoma Bank (DSHB)	1D4B
LAMP2	IHC	1:100	Rat	DSHB	ABL-93
Ki-67	IHC	1:200	Rabbit	Thermo Scientific	RM-9106-S1
Rat IgG, HRP conjugated	IHC	1:100	Goat	Jackson Laboratory	112-035-003
Rabbit IgG, biotinylated	IHC	1:200	Goat	Vector Laboratories	PK-6101
Rabbit IgG, biotinylated	IHC	1:200	Goat	Vector Laboratories	BA-1000

Table S2 List of cancer samples in the multicancer tissue microarrays, including the H-scores for the intensity of RAB24 immunohistochemical staining. (*Excel file*)

Table S3 List of human pancreatic neuroendocrine tumour samples included in the tissue microarray. The table includes H-scores for the intensity of RAB24 immunohistochemical staining in samples derived from the tumour centre, tumour edge, and normal pancreatic tissue. For each patient, up to eight cores per sample type were included in the microarray. The reported H-scores represent the average values for cases with more than one core per region. Sample types not available for a given patient are indicated as N/A (not available). (*Excel file*)

Table S4 List of brain cancer and normal brain tissue samples included in the central nervous system (CNS) tissue microarray (CNS2081a, TissueArray.Com). The table includes patient information and the corresponding H-scores for RAB24 immunohistochemical staining. For each patient, duplicate tissue cores were included in the microarray, and the reported H-scores represent the average of the values determined for both cores. (*Excel file*)

Table S5 List of neuroblastoma and peripheral nerve tissue samples included in the neuroblastoma tissue microarray (NB642d, TissueArray.Com). The table includes patient information and the corresponding H-scores for RAB24 immunohistochemical staining. For each patient, duplicate tissue cores were included in the microarray, and the reported H-scores represent the average of the values determined for both cores. INSS, International Neuroblastoma Staging System. (*Excel file*)

Table S6 sgRNAs used for the creation of Neuro-2a Rab24 knockout cells and HeLa RAB24 knockout cells.

Target location	Genomic location	Target sequence
Mouse Rab24 exon 1	chr13:55321527	GCAGCGCGTGGACGTTAAGG
Mouse Rab24 exon 1/intron1	chr13:55321624	GGTGCACTCACGTTCTGATA
Human RAB24 exon 5	chr5:177302424	AGTCCACACGTCGACGCCTC
Human RAB24 exon 3	chr5:177302780	CCGATAGTAGATTCTACTCA

Table S7 PCR primers used to confirm successful deletion of *Rab24* gene in Neuro-2a and *RAB24* gene in HeLa cells.

Target	Genomic location	Direction	Sequence
Mouse Rab24 promotor (p1)	chr13:55321669-55321688	Forward	GGACCCTGAAGTGGGTGAG
Mouse Rab24 intron 5 (p2)	chr13:55320655-55320677	Reverse	GGACGTCAAGGTGGTGATG
Human RAB24 intron 2 (p3)	chr5:177302955-177302977	Forward	TCATTCCTGAGGAGGTGTAGGT
Human RAB24 intron 5 (p4)	chr5:177302286-177302308	Reverse	AGGAAGACCTCCTAGAGCACCT
